# Molecular insights into type I interferon suppression and enhanced pathogenicity by species B human adenoviruses B7 and B14

**DOI:** 10.1128/mbio.01038-24

**Published:** 2024-06-28

**Authors:** Drayson Graves, Nikolas Akkerman, Lauren Fulham, Rafe Helwer, Peter Pelka

**Affiliations:** 1 Department of Microbiology, University of Manitoba, Winnipeg, Manitoba, Canada; 2 Department of Medical Microbiology and Infectious Diseases, University of Manitoba, Winnipeg, Manitoba, Canada; McMaster University, Hamilton, Ontario, Canada

**Keywords:** adenovirus, interferon, transcription, RuvBL1, RuvBL2

## Abstract

Human adenoviruses (HAdVs) are small DNA viruses that generally cause mild disease. Certain strains, particularly those belonging to species B HAdVs, can cause severe pneumonia and have a relatively high mortality rate. Little is known about the molecular aspects of how these highly pathogenic species affect the infected cell and how they suppress innate immunity. The present study provides molecular insights into how species B adenoviruses suppress the interferon signaling pathway. Our study shows that these viruses, unlike HAdV-C2, are resistant to type I interferon. This resistance likely arises due to the highly efficient suppression of interferon-stimulated gene expression. Unlike in HAdV-C2, HAdV-B7 and B14 sequester STAT2 and RNA polymerase II from interferon-stimulated gene promoters in infected cells. This results in suppressed interferon- stimulated gene activation. In addition, we show that RuvBL1 and RuvBL2, cofactors important for RNA polymerase II recruitment to promoters and interferon-stimulated gene activation, are redirected to the cytoplasm forming high molecular weight complexes that, likely, are unable to associate with chromatin. Proteomic analysis also identified key differences in the way these viruses affect the host cell, providing insights into species B-associated high pathogenicity. Curiously, we observed that at the level of protein expression changes to the infected cell, HAdV-C2 and B7 were more similar than those of the same species, B7 and B14. Collectively, our study represents the first such study of innate immune suppression by the highly pathogenic HAdV-B7 and B14, laying an important foundation for future investigations.

**IMPORTANCE:**

Human adenoviruses form a large family of double-stranded DNA viruses known for a variety of usually mild diseases. Certain strains of human adenovirus cause severe pneumonia leading to much higher mortality and morbidity than most other strains. The reasons for this enhanced pathogenicity are unknown. Our study provides a molecular investigation of how these highly pathogenic strains might inactivate the interferon signaling pathway, highlighting the lack of sensitivity of these viruses to type I interferon in general while providing a global picture of how viral changes in cellular proteins drive worse disease outcomes.

## INTRODUCTION

Human adenoviruses are a large family of DNA viruses belonging to the *Adenoviridae* family of the *Mastadenovirus* genus (1). These non-enveloped and icosahedral viruses commonly infect the airway, the gut, the eye, and the urinary tract (1). Generally, most infections are mild and clear on their own, with minimal effects on the host (1). However, certain strains of HAdVs can cause severe infections in the very young, the elderly, the immunocompromised, and sometimes healthy individuals (2–4). In particular, species B HAdVs are often associated with more severe disease, including types B7 and B14 (5–7). Despite the severity of the disease caused by these strains, little is known about the molecular mechanisms underpinning the disease outcomes.

There have been a number of deadly HAdV species B outbreaks that resulted in significant mortality, particularly of children (4). Among these was an outbreak at a children’s care home in New Jersey in 2018 (8). During this outbreak, 11 children succumbed to the infection out of 34 who became ill. This outbreak was one of two serious outbreaks of HAdV that caused deaths in 2018, the other outbreak occurred at the University of Maryland killing one student while making 40 students seriously sick (8). Other such outbreaks have been reported in the past, all of which have resulted in fatalities, some in immune-compromised individuals such as another outbreak at a long-term children’s care facility in Illinois in 2005 (9), but others in relatively healthy individuals such as an outbreak in a substance abuse rehabilitation facility in New Jersey in 2017 (10), and an outbreak in 2005 that began in Oregon with a high mortality rate of 18% and hospitalization rate of 76% (11). New and emerging strains of HAdV have been circulating in populations with high morbidity and mortality rates, such as variant HAdV-E4 in Hong Kong (12) and HAdV-B14a contributing to deaths in 2007 and 2009 (13). Clearly, these are dangerous pathogens and our lack of understanding of how they suppress innate immunity leaves a significant knowledge gap in elucidating their pathogenicity.

Activation of interferon-stimulated genes (ISGs) occurs rapidly after interferon (IFN) stimulation and relies on recruitment of the transcription factor complex IFN-stimulated gene factor 3 (ISGF3) to promoters, which happens *via* the canonical JAK-STAT pathway (14, 15). IFN stimulation leads to the nuclear translocation of ISGF3, its binding to the IFN-stimulated response element (ISRE), and recruitment of chromatin re-modelers and the RNA polymerase II complex to the promoter, driving ISG activation (16, 17). Suppression of IFN signaling is a key aspect of evading innate immunity by viral pathogens, including HAdVs (1). Indeed, HAdVs have evolved in multiple ways by which they suppress the activation of ISGs to drive viral replication (18). Beyond the well-established role of the virus-associated non-coding RNAs VAI and VAII in suppression of the interferon pathway (19, 20), much focus has been on E1A, which targets the interferon pathway using multiple mechanisms. Specifically, studies of E1A function have elucidated the numerous ways by which E1A is involved in the suppression of innate immunity *via* interactions with cellular regulators including STAT1 (21), FoxK & DCAF7 (22), and RuvBL1 (23). In addition, E4orf3 is known to trap ISGs in a heterochromatin complex, preventing their activation (24). Lastly, studies have implicated E1B-55k protein in the modulation of the innate immune pathway (25).

Limited literature exists on the effects and impact of IFN on species B HAdVs. Previously, it was shown that IFN-β had only a very limited impact on the ability of HAdV-B3 to replicate, whereas IFN-γ had no effect at all (26). Whereas an earlier study (27) showed that HAdV-B3 is significantly inhibited in its replication only by a co-treatment with anti-HAdV-B3 antibody and IFN-β or IFN-γ, but not by IFN treatment alone. Furthermore, a study comparing the sensitivity of different HAdVs strains found that HAdV-C5 is slightly more sensitive to IFN-γ than HAdV-B3 and B7, with the latter being the most resistant (28). Our recent work into HAdV-B7 identified ARGLU1 as a binding partner of E1A (29), linking ARGLU1 to DNA damage response and transcriptional repression.

Our previous work investigated the molecular mechanism of IFN suppression by HAdV-C5 (23). In particular, we observed that E1A of HAdV-C5 interacts with the AAA+ DNA helicase RuvBL1/Pontin to suppress the activation of ISGs during infection. Therefore, we wanted to determine whether RuvBL1 is also a target for the more highly pathogenic HAdV-B7 and B14. Interestingly, we observed that HAdV-B7 and B14 were largely unaffected by the presence of type I IFNs prior to infection, unlike HAdV-C2. We also determined that E1As of HAdVs B7 and B14 bind to RuvBL1 and RuvBL2/Reptin, with higher affinity for RuvBL2 than E1A from HAdV-C2. Importantly, suppression of ISG activation relied on the inhibition of recruitment of Signal Transducer and Activator of Transcription 2 (STAT2) and RNA polymerase II to ISG promoters. The proteomic analysis also revealed significant differences in how these pathogens affected the infected cell, revealing pathways that may contribute to enhanced pathogenicity of species B viruses. This study represents an important advancement in understanding mechanisms underlying the severity of disease caused by HAdV-B7 and B14.

## RESULTS

### HAdVs B7 and B14 are minimally affected by IFN

**Fig 1:**
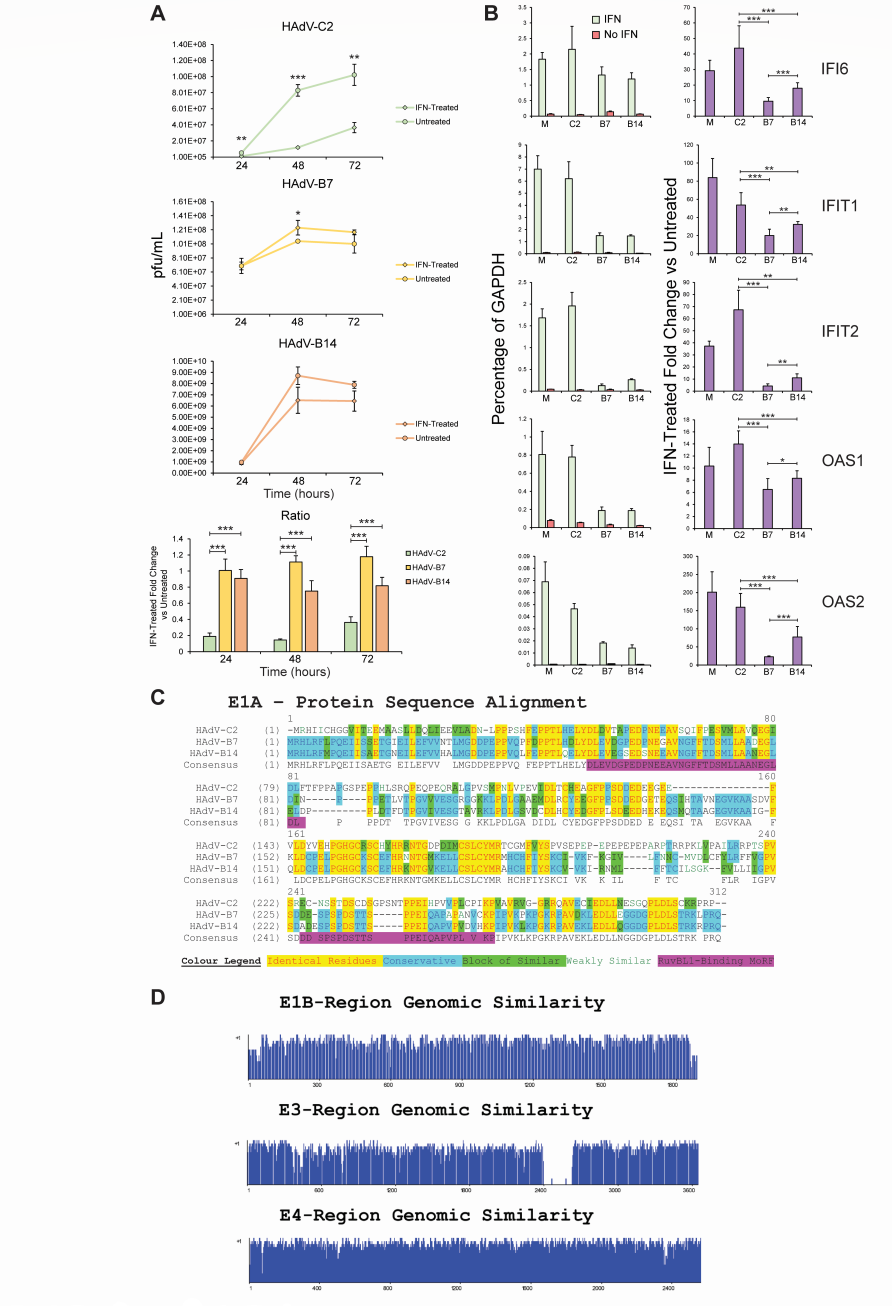
Notable gaps in or around the RuvBL1 binding areas of E1A accompany a difference in susceptibility to IFN. (**A**) HAdVs B7 and B14 grow better in IFN-treated cells than HAdV C2. A549 cells treated 16 hours prior with IFN α-2A were infected with HAdV strains C2, B7, and B14, and harvested 24, 48, and 72 hours after infection. Viral titer was measured as pfu/mL *via* plaquing assay. The bar graph represents the same results as a ratio of IFN-treated samples to untreated samples. (**B**) ISG expression is severely hampered in cells infected with HAdVs B7 and B14 compared to those infected with HAdV-C2. A549 cells were treated with IFN α-2A 16 hours after infection with indicated HAdV strains. Eight hours later, the cells were harvested, RNA extracted, and cDNA generated. Interferon-stimulated gene expression was analyzed *via* real-time qPCR. Results were normalized to percentage expression of the housekeeping gene *GAPDH* (left panel) and as a ratio of IFN-treated over untreated (right panel). M: mock infected, C2: HAdV-C2, B7: HAdV-B7, B14: HAdV-B14. (**C**) Strong conservation is present within the RuvBL1-binding site in HAdV-C2, B7, and B14 E1A. Indicated E1A protein sequences acquired from Genbank were aligned *via* AlignX software. Residues are color-coded in accordance with the supplied color legend. (**D**) General interspecies similarity levels in E1B, E3, and E4 early transcription regions. E1B, E3, and E4 genomic regions of HAdV species C2, B7, and B14 were aligned *via* AlignX software. Histograms represent overall sequence similarity. Statistical analysis was performed by unpaired student’s *t*-test. One asterisk represents a *P*-value of <0.05, two represent <0.01, and three represent <0.001; *n* = 3.

We wanted to determine the sensitivity of HAdV-B7 and B14 to interferon. Previous studies with HAdV-C5, strain *dl*309, showed that this virus is not sensitive to IFN α treatment in 293 or KB cells and grows to roughly equivalent titers regardless (20). To determine whether species B viruses are sensitive to IFN α treatment, A549 cells were treated with IFN α-2a for 16 hours prior to infection with HAdV-C2, B7, or B14. Viral titers were assayed at 24, 48, and 72 hours after infection (Fig. 1A). Interestingly, we observed that HAdV-C2 was significantly affected by pre-treatment with IFN by nearly one log growth reduction at 48 and 72 hours after infection, while HAdV-B7 and B14 were minimally affected (Fig. 1A). When we plot the ratios of IFN-treated over untreated titers, the difference between the viruses is highly significant (Fig. 1A). Interestingly, HAdV-B14 replicated much more rapidly as compared to the other strains, reaching relatively high titers after 24 hours of infection. Titers of HAdV-C2 were reduced to approximately 20% of the untreated titers with IFN treatment, whereas titers of HAdV-B7 were unaffected, while overall titers of HAdV-B14 reduced only slightly to about 80% of the untreated levels (Fig. 1A). We also investigated whether the appearance of the cytopathic effect (CPE) was affected by IFN. Figure S1 shows that there were no observable effects of IFN treatment on the course of infection, a similar phenotype was also observed in primary IMR-90 cells (Fig. S2A). Similarly, expression of viral genes and proteins was not significantly affected by IFN treatment with these viruses (Fig. S3 and S4, respectively). A possibility exists that IFN inhibits virus entry into the cell. We tested this by treating the cells with IFN prior to infection followed by infection and immunofluorescence for viral particles, all viruses showed relatively similar infection efficiency regardless of IFN (Fig. S5). We also investigated whether the presence of IFN can affect plaquing on A549 cells (Fig. S6). Interestingly, although no effect was observed for HAdV-C2 and B7, a large plaque phenotype was observed in HAdV-B14. We later determined this to likely be caused by apoptosis of IFN-treated and HAdV-B14-infected A549 cells based on cellular genomic DNA fragmentation (data not shown). Collectively, these results demonstrate that while HAdV-C2 is sensitive to IFN treatment, HAdV-B7 and B14 are minimally affected.

### Species B HAdVs can efficiently suppress ISG expression

Our previous studies in HT1080 cells (23) have shown that HAdV-C5 is reliant on RuvBL1 for efficient suppression of ISGs during infection. We also hypothesized that one reason behind the enhanced pathogenicity of species B HAdVs is their efficient ability to shut down the IFN pathway and suppress the activation of ISGs. To investigate how ISGs are affected by HAdV-B7 and B14 infection in cells treated with IFN, we infected A549 cells for 16 hours, followed by IFN α-2a treatment, and RNA collection 8 hours later, as we have done previously (23). We determined the expression levels of *IFI6*, *IFIT1*, *IFIT2*, *OAS1*, and *OAS2* (Fig. 1B). Under these conditions, HAdV-C2 was significantly less efficient in suppressing ISG expression as compared to HAdV-B7 and B14, which were able to efficiently suppress the transcription of all ISGs with *IFI6* being suppressed the least. Importantly, the gene expression ratios of IFN treated to untreated cells showed that species B viruses are much more efficient at suppressing the expression levels of these ISGs following infection and IFN treatment (Fig. 1B). Similarly, suppression of ISGs was much more efficient by HAdV-B7 in primary IMR-90 cells as compared to HAdV-C2 (Fig. S2B). Suppression of ISG activation in A549 cells by HAdV-C2 was somewhat different than what we have previously observed in HT1080 cells with the closely related HAdV-C5 (23). This is likely due to cell type differences and timing variation in infection and IFN application and is unlikely caused by differences in E1A protein sequence between the two types as the three amino acid variances lie outside of the RuvBL1-binding region.

### Alignment of the E1A, E1B, E3, and E4 regions of HAdVs C2, B7, and B14

To investigate the potential mechanism of how species B HAdVs can efficiently suppress IFN response, we examined sequence alignments of the regions of HAdV genome with known links to innate immunity suppression: E1A, E1B, E3, and E4; with our main focus on E1A (Fig. 1C and D). Sequence alignments of the largest E1A proteins from HAdV-C2, B7, and B14 showed a relatively high level of similarity, with the region in the second exon that is known to bind to RuvBL1 having a small deletion found in species B viruses versus species C viruses. Modulation of RuvBL1 function, and therefore ISG suppression, may be affected by this deletion and this became the focus of the current study. Analysis of the other early regions found no large differences in E1B, while an insertion present in the E3 region of only HAdV-B7 was observed. The function of this insertion is unknown, but the putative protein product from this region is predicted to be glycosylated with a transmembrane domain and merits future investigation. The E4 region was relatively highly conserved between the three viruses examined, with no unique open reading frames present in any of them. Because of the involvement of RuvBL1 in ISG suppression by HAdV-C5 that we reported earlier (23), we decided to focus our initial investigation on the RuvBL family.

### Species B HAdVs E1A bind efficiently to RuvBL1 and RuvBL2

**Fig 2:**
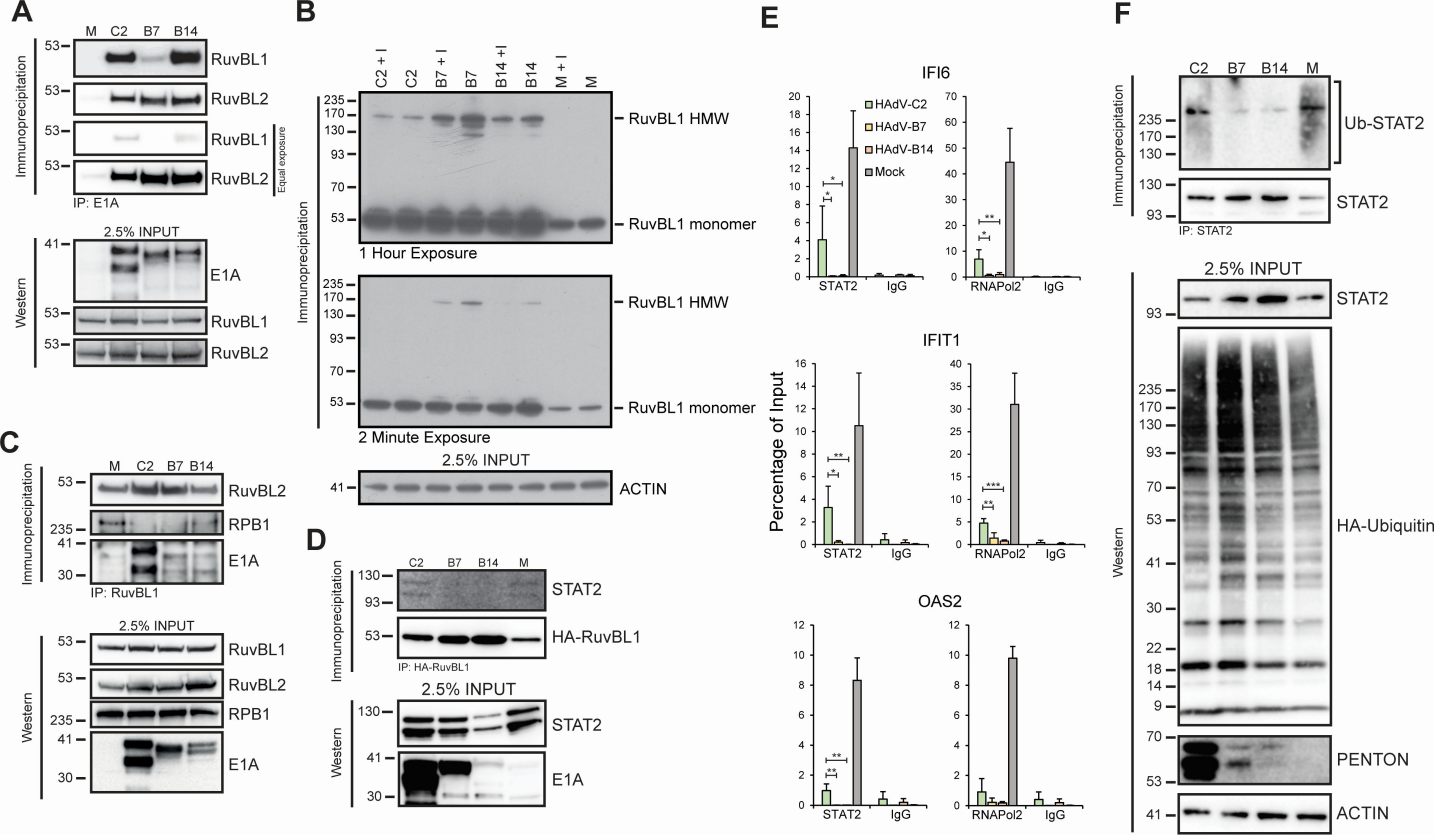
RuvBL1 complex formation during infection accompanies decreased RuvBL1-E1A and -STAT2 binding affinity and decreased transcription factor recruitment to ISG promoters. (**A**) HAdV E1As bind to RuvBL1 and RuvBL2. A549 cells were infected with indicated viral strains for 24 hours, lysed, and indicated proteins were immunoprecipitated. Precipitates were resolved on SDS gel, transferred to the PVDF membrane, and blotted with indicated antibodies. M: mock infected, C2: HAdV-C2, B7: HAdV-B7, B14: HAdV-B14. (**B**) HAdV-B7 and HAdV-B14 induce complex formation of RuvBL1 in infected cells. A549 cells were transfected with HA-tagged RuvBL1 expression vector and infected with indicated viruses for 24 hours. Eight hours before harvest, indicated samples were treated with IFN α-2A. Cells were cross-linked with formaldehyde, harvested, and lysed, and HA-RuvBL1 was precipitated using 12CA5 anti-HA mouse antibody. Precipitates were resolved on precast, 4%–12% gradient SDS gel, transferred to the PVDF membrane, and blotted with the indicated antibodies (RuvBL1 was blotted with a rat anti-HA antibody different from that used to precipitate it); HMW, high molecular weight. (**C**) HAdV infection disrupts RuvBL1/RNA Pol II interaction. A549 cells were infected with indicated viral strains for 24 hours, lysed, and indicated proteins were immunoprecipitated. Precipitates were then resolved on SDS gel, transferred to the PVDF membrane, and blotted with the indicated antibodies. RPB1: RNA Polymerase II. (**D**) HAdV-B7 and B14 disrupt STAT2/RuvBL1 interaction. A549 cells were transfected with HA-tagged RuvBL1 expression vector and infected with the indicated HAdV strains 24 hours later. Sixteen hours after infection, IFN α-2A was applied. After 8 more hours, cells were harvested and lysed, and HA-tagged RuvBL1 was precipitated *via* 12CA5 anti-HA antibody. Samples were resolved on SDS gel, transferred to PVDF membrane, and blotted with the indicated antibodies. (**E**) Recruitment of STAT2 and RNA polymerase II to ISG promoters is dramatically reduced in HAdV-B7 and HAdV-B14 compared to HAdV-C2. A549 cells were treated with IFN α-2A 20 hours after infection with the indicated HAdVs, and 4 hours later (24 hours after infection), cells were cross-linked, harvested, and indicated proteins were immunoprecipitated. Cross-linking was reversed, DNA was isolated, and relative levels were analyzed *via* qPCR. Statistical analysis was performed *via* unpaired student’s *t*-test. One asterisk signifies a *P*-value of <0.05, two represent <0.01, and three indicate <0.001; *n* = 2. (**F**) Ubiquitination of STAT2 is reduced in HAdV-B7 and B14-infected cells. HT1080 cells were transfected with the HA-ubiquitin expression vector and subsequently infected with the indicated viruses for 24 hours at an MOI of 10. Cells were lysed and immunoprecipitations were carried out for STAT2 in RIPA buffer as described. Immunoprecipitates were then resolved on a 4%–12% gradient SDS gel and blotted for the indicated proteins. PENTON was used to indicate infection, note that higher levels of PENTON in C2 do not reflect higher protein expression in this virus, but rather the sensitivity of the anti-PENTON antibody for C2, for which it was raised, is much higher than for B7 and B14.

Sequence alignment showed strong conservation within the region of E1A that binds to RuvBL1 (Fig. 1C). We hypothesized that species B HAdVs can bind strongly to RuvBL1 to suppress activation of ISGs. To test this hypothesis, we investigated the interaction of RuvBL1, RuvBL2, and E1As from HAdV-C2, B7, and B14 (Fig. 2A) *via* immunoprecipitation. All E1As interacted with RuvBL1, with HAdV-C2 and B14 showing the strongest interaction while HAdV-B7 showed the weakest, but still readily detectable, interaction. Interestingly, all serotypes showed much stronger binding to RuvBL2 than RuvBL1, with species B types 7 and 14 showing the strongest binding (Fig. 2A).

### HAdV-B7 and B14 drive RuvBL1 into high molecular weight complexes

RuvBL1 is known to form high molecular weight complexes with itself and RuvBL2, as well as with other proteins (30). Importantly, our results (Fig. 2A) show that E1As from the serotypes tested bound stronger to RuvBL2 than RuvBL1. A potential mechanism of ISG suppression is the sequestration of the RuvBL proteins into complexes with impaired or altered functions. To test this possibility, we performed a cross-linking immunoprecipitation where cells were crosslinked with 1% formaldehyde prior to lysis to stabilize protein complexes, as we have done in the past (31). We then performed immunoprecipitation for RuvBL1 and detected RuvBL1 complexes (Fig. 2B). Infection with HAdVs caused high molecular weight complexes to form, with HAdV-B7 and B14 showing the highest degree of complex formation and HAdV-C2 showing the least. No complexes were detected in mock-infected cells, although this may be due to lower overall expression of transfected RuvBL1 in uninfected cells. IFN treatment had a minimal effect on complex formation. The reduced expression of transfected RuvBL1 in uninfected cells is likely due to a lack of E1A, which is known to activate the CMV enhancer used to drive HA-RuvBL1 expression (32). Nevertheless, these results clearly demonstrate that RuvBL1 is retained or stabilized in high molecular weight complexes to a greater degree with species B infection versus HAdV-C2, suggesting that this complex may impair ISG expression.

### Infection with HAdV disrupts the interaction between RuvBL1 and RNA polymerase II

The formation of high molecular weight complexes induced by HAdV infection and involving RuvBL1 observed in Fig. 2B suggests that these may be non-functional. The binding of RuvBL1 and RuvBL2 to RNA polymerase II is important for ISG activation (33), while RuvBL2 also serves as an important factor required for licensing of RNA polymerase II at promoters (34). Therefore, we wanted to determine whether infection with HAdVs affects the ability of RuvBL1 to interact with RNA polymerase II or RuvBL2 (Fig. 2C). Indeed, infection with HAdV-C2 and HAdV-B7 seems to greatly reduce the binding of RuvBL1 to RNA polymerase II, while infection with HAdV-B14 significantly reduces it. Furthermore, interaction between RuvBL1 and RuvBL2 is also affected, with HAdV-C2 and B7 enhancing this association, while HAdV-B14 has minimal effects (Fig. 2C). Together, these results demonstrate that HAdVs differentially affect the interaction of RuvBLs with themselves and RNA polymerase II.

### Interaction between STAT2 and RuvBL1 is disrupted in HAdV-B7 and B14-infected cells

STAT2 is a critical component of ISGF3 and is required for ISG activation upon IFN α stimulation (35, 36). STAT2 was also previously shown to interact with RuvBL1 and RuvBL2 during ISG activation (33). We next determined whether the RuvBL1 and STAT2 interaction was affected by HAdV infection, which represents a potential mechanism of ISG suppression by these viruses. Immunoprecipitation of RuvBL1 from infected A549 cells showed a loss of interaction between RuvBL1 and STAT2 in cells infected by HAdV-B7 and B14, but not from mock-infected cells, or cells infected with HAdV-C2 (Fig. 2D). These results suggest that STAT2 function is impaired in HAdV-B7 and B14-infected cells more efficiently than by HAdV-C2.

### Recruitment of STAT2 and RNA polymerase II to ISG promoters is impaired in HAdV-infected cells

Recruitment of STAT2 and RNA polymerase II to ISG promoters leads to rapid activation of ISGs after IFN treatment [reviewed by (37)]. Since we observed reduced interaction between RuvBL1 and STAT2 (Fig. 2D) and reduced interaction between RNA polymerase II and RuvBL1 (Fig. 2C) in infected cells, we next determined whether recruitment of STAT2 and RNA polymerase II is differentially affected by HAdV-C2, B7, and B14. Chromatin immunoprecipitation (ChIP) was performed on IFN-treated and infected A549 cells 24 hours after infection (Fig. 2E). We investigated promoter occupancy of STAT2 and RNA polymerase II on *IFI6*, *IFIT1*, and *OAS2* promoters. STAT2 and RNA polymerase II were significantly reduced at the ISG promoters examined versus mock-infected cells. Significantly, the levels of STAT2 and RNA polymerase II in HAdV-B7 and B14-infected cells were at, or nearly at, the level of background, whereas it was significantly above background in HAdV-C2-infected cells. Similar findings were observed in primary IMR-90 cells infected with HAdV-C2 or HAdV-B7 (Fig. S2C). We also investigated the recruitment of RuvBL1 to ISG promoters during virus infection, overall RuvBL1 was reduced at ISG promoters with all viruses tested as compared to mock-infected cells, but there were no significant differences observed between HAdV-C2, B7, and B14 (Fig. S7).

One possibility that could explain the reduced recruitment of STAT2 to ISGs would be a reduction in protein levels, potentially through proteasome-mediated degradation, as has been previously reported for STAT2 (38) and reviewed (39). To investigate this possibility, we wanted to determine whether infection with HAdVs differentially affects STAT2 levels and ubiquitination. To determine this, HT1080 cells were transfected with an HA-tagged ubiquitin expression vector and infected with HAdVs C2, B7, and B14 (Fig. 2F). STAT2 was subsequently immunoprecipitated and ubiquitinated STAT2 was detected with an anti-HA antibody. Interestingly, we observed that STAT2 ubiquitination is reduced in species B-infected cells as compared to species C2 (Fig. 2F), with total levels of STAT2 also slightly higher in the cells infected with species B viruses as compared to mock-treated or HAdV-C2-infected cells.

Recently, it was shown that GCN5 and BRD2 are required for the eviction of H2A.Z from nucleosomes and higher ISG expression (40). Therefore, we investigated whether H2A.Z, histone H3 lysine 4 tri-methyl [which co-localizes with H2A.Z on promoters (41)] and total histone H3 are present differentially at ISG promoters (Fig. S8). We found no significant differences in the presence of these histones at ISGs versus the *ACTB* promoter, although we did observe an overall increase in histone density at all promoters tested (Fig. S8), which may be caused by E4orf3-driven heterochromatinization as previously reported (42, 43). Knockdown of H2A.Z or GCN5 *via* siRNA also did not have an appreciable effect on ISG suppression in infected cells (Fig. S9).

Collectively, these results demonstrate that species B HAdVs are highly efficient at inhibiting the recruitment of STAT2 and RNA polymerase II to cellular ISG promoters but are largely unaffected by reduced levels of H2A.Z or GCN5.

### RuvBL1 or RuvBL2 knockdown has minimal effects on ISG suppression by HAdV-B7 and B14

**Fig 3:**
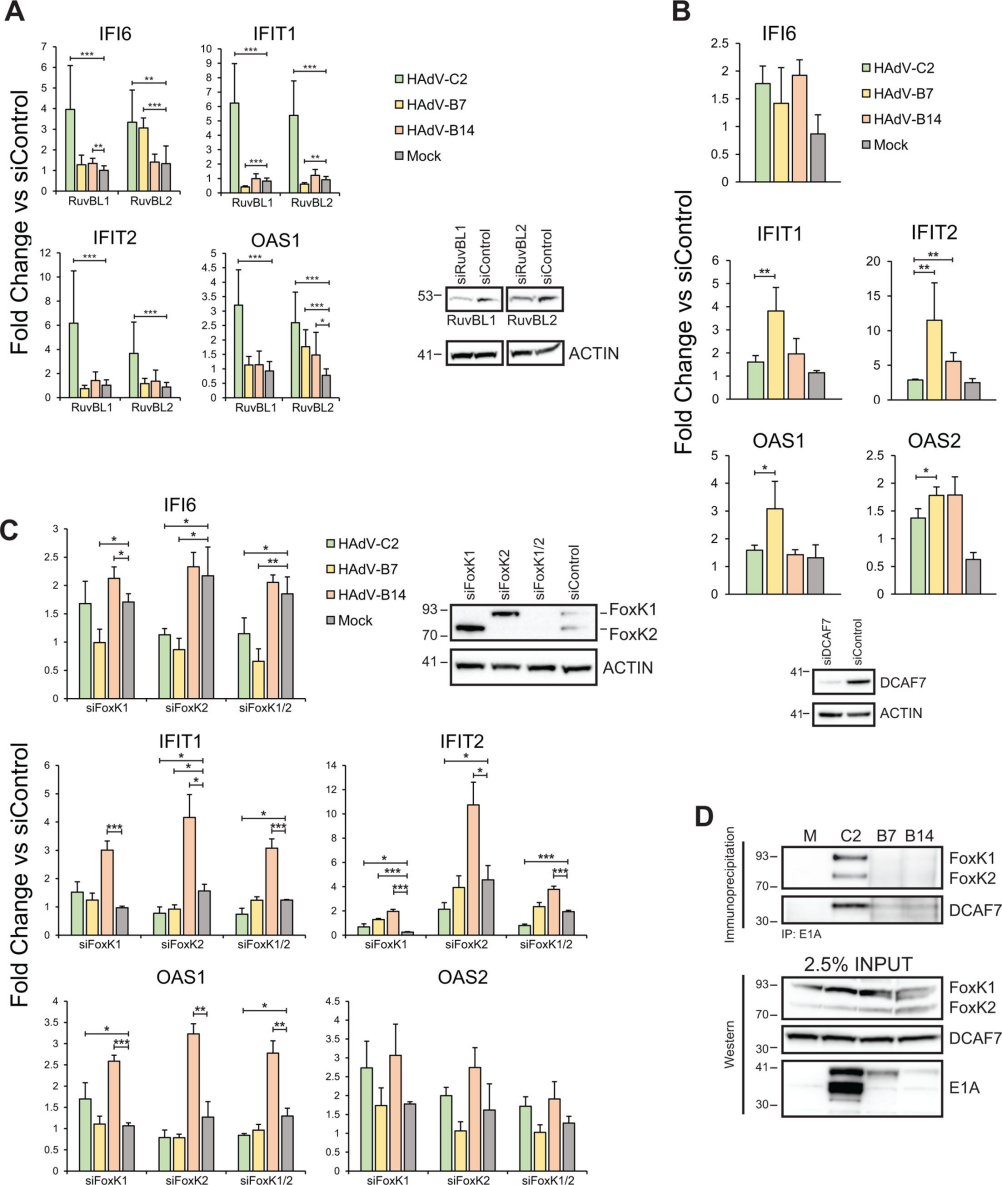
Viral ISG suppression relies on the presence of different, species-specific host proteins. A549 cells were treated with siRNA to knock down RuvBL1 or RuvBL2 (**A**), DCAF7 (**B**), and FoxK1/FoxK2 (**C**), as indicated, for 72 hours. Cells were then infected with indicated HAdV strains and treated with IFN 16 hours later. After 8 more hours, RNA was extracted, cDNA generated, and gene expression measured *via* qPCR. Results are presented as fold change versus cells treated with control siRNA. (**D**) Species C HAdV E1A interacts more strongly with FoxK and DCAF7 than species B. A549 cells were infected with indicated viral strains for 24 hours, lysed, and indicated proteins were immunoprecipitated. These were then resolved on SDS gel, transferred to the PVDF membrane, and blotted with indicated antibodies. M: mock infected, C2: HAdV-C2, B7: HAdV-B7, B14: HAdV-B14. Statistical analysis was performed by unpaired student’s *t*-test. One asterisk represents a *P*-value of <0.05, two represent <0.01, and three represent <0.001; *n* = 3.

We have previously observed that knockdown of RuvBL1 *via* siRNA transfection impaired the ability of HAdV-C5 to efficiently suppress ISG activation (23). Knockdown of RuvBL1 had no significant effect on the ability of HAdV-B7 and B14 to suppress ISGs while enhancing ISG activation in HAdV-C2 infected cells, consistent with our previous observations (Fig. 3A). Knockdown of RuvBL2 had similar effects on the ability of HAdV-C2 to suppress ISGs, that is the virus was not as efficient in ISG suppression when RuvBL2 was knocked down, similarly to what was observed for RuvBL1 (Fig. 3A). Interestingly, the results were more mixed for species B viruses. Specifically, suppression of *IFI6* and *OAS1* was impaired by HAdV-B7 when RuvBL2 was knocked down, but not of *IFIT1* nor *IFIT2*, while only *OAS1* expression in HAdV-B14 infection was affected by RuvBL2 knockdown. These results suggest that while a major route for ISG suppression for HAdV-C2 is through RuvBL1 and RuvBL2, this is largely not the case for HAdV-B7 and B14.

Moreover, recently three additional E1A-binding proteins have been implicated in ISG suppression by HAdV species C, FoxK1&2 (collectively FoxK), and DCAF7 (22). We therefore investigated the impact of these factors on the ability of HAdVs to suppress ISG activation (Fig. 3B and C). Knockdown of DCAF7 had a relatively minimal effect on suppression of *IFI6*, *IFIT1*, *OAS1*&*2* while affecting *IFIT2* slightly more with HAdV-B7 infection. Knockdown of either FoxK1 or 2, or both, had similar effects to what we observed for DCAF7. Investigation of whether these factors bind to species B E1A (Fig. 3D) showed no detectable binding for FoxK and minimal binding by DCAF7. Together, these results suggest that FoxK has a major impact on ISG suppression by species B HAdVs, while knockdown of DCAF7 had a significant effect on the ability of HAdV-B7 to suppress some ISGs as compared to HAdV-C2.

### RuvBL1 and RuvBL2 are retained in the cytoplasm in cells infected by species B HAdVs

**Fig 4:**
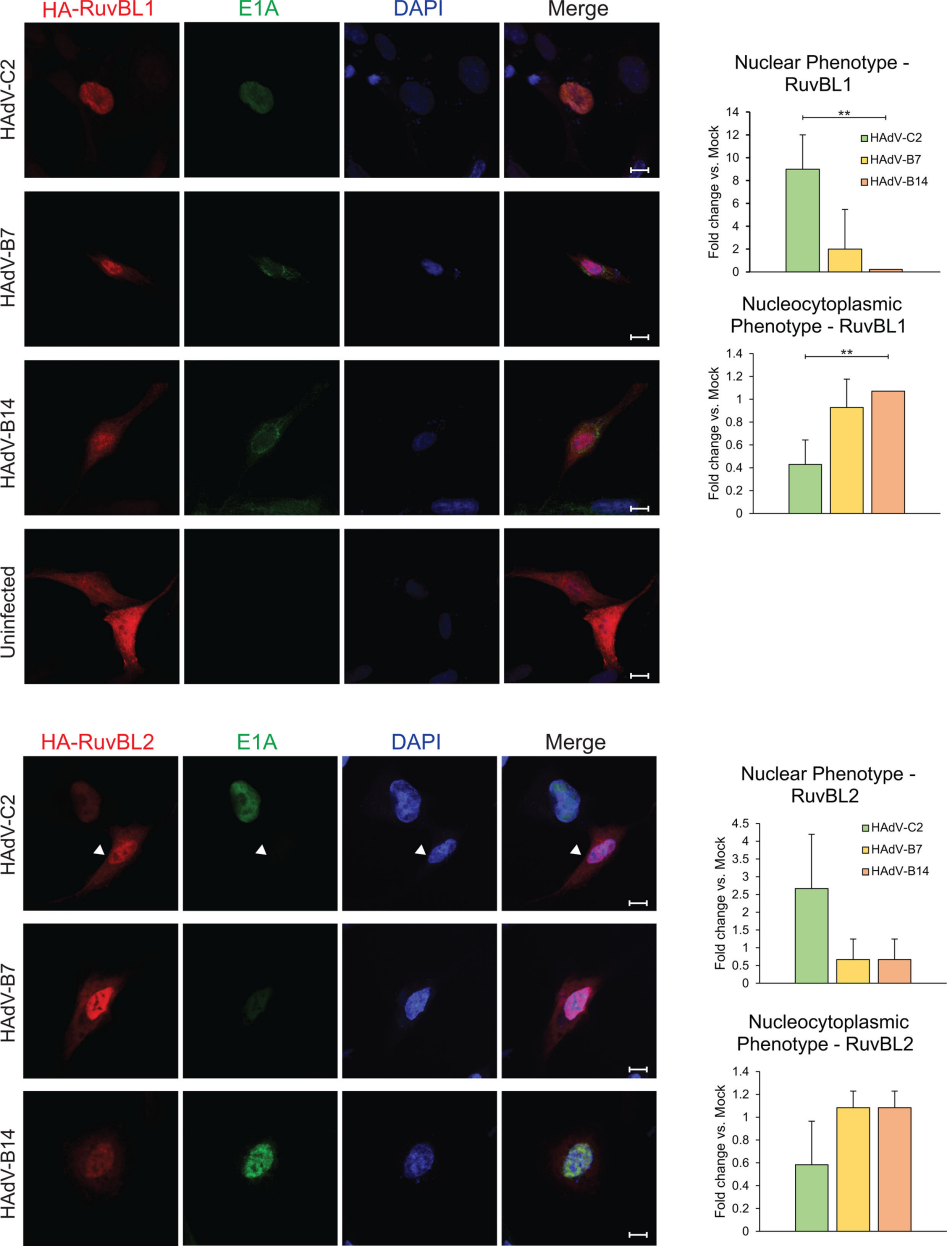
RuvBL1 and RuvBL2 are unable to completely localize to the nucleus in cells infected with HAdV-B7 and HAdV-B14. HT-1080 cells were seeded onto chamber slides and infected with indicated viral strains. After 16 hours, IFN was applied and cells were fixed and permeabilized 8 hours later. Antibodies were then applied as indicated and cells were imaged on a Zeiss LSM700 laser confocal microscope. Carats show uninfected cells as internal controls; scale bar represents 5 μm. Bar charts show the relative frequency of the observed phenotypes with two asterisks representing a *P*-value of <0.01; *n* = 5 random fields of view.

Reduced localization to ISG promoters of STAT2 and RNA polymerase II may be a consequence of the sequestration of RuvBL1 and RuvBL2 into potentially non-functional complexes, as observed in Fig. 2B. These complexes may also have altered sub-cellular localization, as we have observed a similar tactic by HAdV toward other cellular proteins [see (44–46)]. To determine the sub-cellular localization of RuvBL1 and RuvBL2, HT1080 cells were infected with HAdVs and imaged using fluorescence confocal microscopy (Fig. 4). In uninfected IFN-treated cells, RuvBL1 and RuvBL2 were nucleocytoplasmic with some nuclear enrichment observed for RuvBL2. In HAdV-C2-infected cells, RuvBL1 and RuvBL2 were found predominantly within the nucleus, with little cytoplasmic protein detectable. Interestingly, in HAdV-B7 and B14-infected cells, there was a distinct nucleocytoplasmic localization of RuvBL1 and RuvBL2. Similarly, when transfected E1A was co-expressed with transfected HA-tagged RuvBL1, HAdV-C2 E1A-transfected cells showed predominantly a nuclear RuvBL1 localization, while cells expressing HAdV-B7 E1A showed a predominantly cytoplasmic or nucleocytoplasmic RuvBL1 staining (Fig. S10A). This observation suggests that E1A alone may be sufficient to alter RuvBL1 sub-cellular localization. However, when we investigated the proportion of RuvBL1/2, on a global scale early in infection, in nuclear and cytoplasmic fractions we did not observe a difference (Fig. S10B), likely due to a relatively low number of cells infected at that stage of infection. Nevertheless, these results suggest that in species B-infected cells, RuvBL1 and RuvBL2 are retained in the cytoplasm more readily than in species C-infected cells, and therefore may be unavailable for promoter recruitment and ISG activation. We also investigated the effects of infection on STAT1 and STAT2 sub-cellular localization (Fig. S10C) but observed no significant differences. Lastly, we investigated whether HAdVs affect the interaction between STAT1/2 and RNA polymerase II (Fig. S11). We observed a reduction in the interaction between the polymerase and STAT2 under infection conditions for all viruses, but no significant differences were induced by species B HAdVs. These results indicate that unlike HAdV-C2, HAdV-B7 and B14 alter the sub-cellular localization of RuvBL1 and RuvBL2, but not STAT1 nor STAT2.

### Proteomic comparison of HAdV-B and HAdV-C

**Fig 5:**
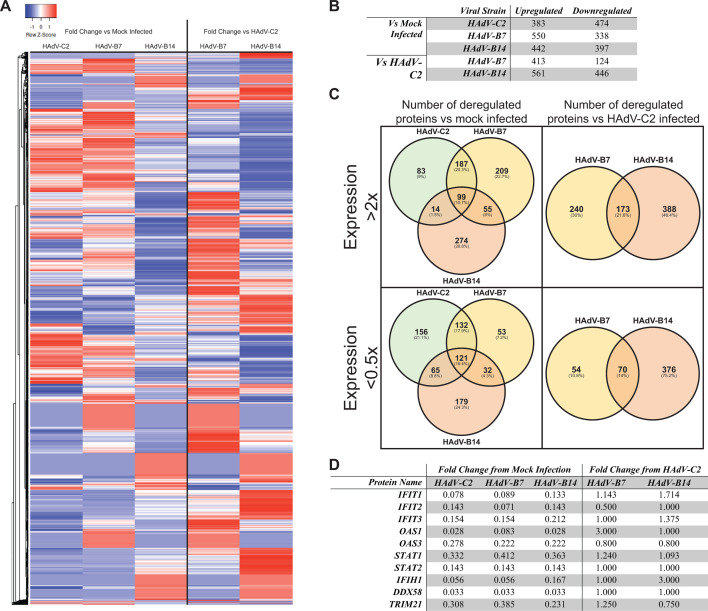
Proteomic analysis of HAdV-B and C infections. A549 cells were infected with HAdV-C2, HAdV-B7, HAdV-B14, or a mock infection as indicated, IFN-treated, and harvested. Protein was extracted and analyzed *via* 2D-LC-MS. (A) Results were broadly visualized *via* heat mapper software. Red indicates upregulation and blue represents downregulation. (B) Numbers of proteins up- and downregulated in HAdV-B and C as compared to uninfected control and HadV-B as compared to HAdV-C. (C) Venn diagrams showing overlap of proteins up- and downregulated in each condition. (D) Selection of notable ISGs and their levels of up- and downregulation.

To investigate the broad-range effects of infection on cellular IFN response, we infected A549 cells with HAdV-C2, B7, and B14, treated them with IFN, and analyzed their proteome *via* shotgun 2D-LC-MS/MS. Results were compared by two metrics: Proteins upregulated or downregulated at least twofold in each infected cell line as compared to an uninfected control and proteins similarly up- or downregulated in HAdV-B7 and B14 as compared to C2 (Fig. 5A). Overall, we observed hundreds of proteins differentially expressed following infection with the three different HAdV types (Fig. 5B). Significant overlap in proteins up- or downregulated was observed between HAdV-B7 and B14, with ~21% commonly upregulated versus HAdV-C2, and ~14% downregulated (Fig. 5C). Between the three types tested, there was ~11% of proteins commonly upregulated and 16% downregulated (Fig. 5C). Unexpectedly, when comparing C2 with B7 we observed a somewhat higher degree of similarity than between B7 and B14, with ~20% of proteins commonly upregulated and ~18% downregulated, as opposed to ~6% upregulated and ~4% downregulated when comparing B7 to B14.

We also investigated the biological processes up- and downregulated by each strain (Tables 1 and 2). HAdV-B7 was particularly efficient at upregulating processes involved in cell RNA metabolism, while HAdV-B14 upregulated energy and biomolecule synthesis pathways. Interestingly, we observed a high degree of downregulation of innate defense pathways by HAdV-B7 including MDA5, T-cell-mediated cytotoxicity, response to type I IFN, and negative regulation of viral genome replication. Conversely, HAdV-B14 was found to suppress ISG15-protein conjugation, NF-κB signaling, and pyroptosis. Furthermore, HAdV-B7 and B14 were found to be better, as compared to HAdV-C2, at suppressing protein dephosphorylation pathways, ubiquitination-mediated protein catabolic processes, and cellular metabolic compound salvage pathways. These observations suggest that HAdV-B7 is able to strongly downregulate a variety of innate immune pathways, whereas HAdV-B14 focuses on IFN-regulated pathways while upregulating energy-producing metabolic processes while inhibiting immune-driven cell death.

**TABLE 1:** Notable biological processes upregulated in HAdV-B7 and HAdV-B14^*a*^

Upregulated biological processes	Fold enrichment in indicated strain		
HAdV-B7	*C2*	*B7*	*B14*
mRNA 5'-splice site recognition (GO:0000395)	NC	14.75	NC
mRNA 3'-splice site recognition (GO:0000389)	NC	14.75	NC
miRNA-mediated gene silencing by inhibition of translation (GO:0035278)	NC	7.37	NC
mRNA 3'-end processing (GO:0031124)	4.82	6.51	NC
rRNA transcription (GO:0009303)	NC	6.45	NC
Multivesicular body organization (GO:0036257)	NC	6.03	NC
HAdV-B14	*C2*	*B7*	*B14*
Positive regulation of mitochondrial RNA catabolic process (GO:0000962)	NC	NC	26.17
Acetyl-CoA biosynthetic process (GO:0006085)	NC	NC	13.08
Viral mRNA export from host cell nucleus (GO:0046784)	NC	NC	13.08
Glutathione transport (GO:0034635)	NC	NC	11.63
Tricarboxylic acid cycle (GO:0006099)	NC	NC	10.31
Proton motive force-driven ATP synthesis (GO:0015986)	NC	NC	8.6
Ribonucleoside triphosphate biosynthetic process (GO:0009201)	NC	NC	6.6
ATP metabolic process (GO:0046034)	NC	NC	5.99
Nucleoside triphosphate metabolic process (GO:0009141)	NC	NC	5.2
HAdV-B7 and HAdV-B14	*C2*	*B7*	*B14*
Transcription elongation-coupled chromatin remodeling (GO:0140673)	NC	14.75	17.44
Regulation of RNA export from nucleus (GO:0046831)	NC	11.06	13.08
mRNA cis splicing, via spliceosome (GO:0045292)	NC	9.22	10.9
Regulation of nucleobase-containing compound transport (GO:0032239)	NC	8.85	10.47
Negative regulation of DNA-templated transcription, elongation (GO:0032785)	NC	7.04	8.33

^*a*^ Biological process gene ontologies were generated from genes upregulated in HAdV species compared to uninfected control. Processes uniquely enriched more than five times in indicated strains were isolated, and pathways of interest are presented here. NC is short for no change. *P*-values associated with presented numbers are all less than 0.01.

**TABLE 2:** Notable biological processes downregulated in HAdV-B7 and HAdV-B14^*a*^

Downregulated biological processes	Fold enrichment in indicated strain		
HAdV-B7	*C2*	*B7*	*B14*
MDA-5 signaling pathway (GO:0039530)	NC	22.29	NC
Response to type I interferon (GO:0034340)	4.82	6.05	4.67
Negative regulation of viral genome replication (GO:0045071)	4.64	5.84	4.5
Positive regulation of T-cell-mediated cytotoxicity (GO:0001916)	NC	5.2	NC
HAdV-B14	*C2*	*B7*	*B14*
ISG15-protein conjugation (GO:0032020)	NC	NC	15.27
Somatic diversification of immune receptors via somatic mutation (GO:0002566)	NC	NC	8.81
Negative regulation of NIK/NF-kappa B signaling (GO:1901223)	NC	NC	5.73
Pyroptosis (GO:0070269)	NC	NC	5.17
HAdV-B7 and HAdV-B14	*C2*	*B7*	*B14*
Negative regulation of protein dephosphorylation (GO:0035308)	4.74	6.5	6.44
Ubiquitin-dependent protein catabolic process via the multivesicular body sorting pathway (GO:0043162)	4.69	5.62	5.57
Cellular metabolic compound salvage (GO:0043094)	NC	5.57	7.16

^*a*^ Biological process gene ontologies were generated from genes downregulated in HAdV species compared to uninfected control. Processes uniquely enriched more than five times in indicated strains were isolated, and pathways of interest are presented here. NC is short for no change. *P*-values associated with presented numbers are all less than 0.01.

## DISCUSSION

In the present study, we set out to determine how HAdV-B7 and B14 differ in their suppression of the type I IFN pathway versus the well-studied HAdVs from species C. Our results show that HAdV-B7 and B14 are minimally affected by pre-exposure of infected cells to IFN, unlike HAdV-C2, which grew significantly worse when cells were pre-treated with IFN (Fig. 1A). This reduction was not due to reduced viral gene and protein expression, as those were unaltered (Fig. S3 and S4), and suggests host-intrinsic inhibition that does not affect viral gene regulation. Our previous work implicated RuvBL1 in E1A-mediated suppression of ISGs (23), a member of the AAA+ family of DNA helicases that also has a close relative, RuvBL2. Therefore, we focused our study on whether some of the differences in how these viruses affect the IFN pathway may be due to differential regulation of RuvBL1/2 function. All E1A proteins from HAdVs tested bound to RuvBL1 and RuvBL2, with significantly stronger binding observed for RuvBL2 than RuvBL1 (Fig. 2A). HAdV-C2 and B7 were also better at inhibiting or disrupting the interaction between RuvBL1/2 and RNA polymerase II (Fig. 2C). Interestingly, recruitment of RNA polymerase II and STAT2 to ISG promoters was inhibited more with species B viruses than HAdV-C2, resulting in significantly greater inhibition of ISG transcription by HAdV-B7 and B14 versus C2 (Fig. 2E). Curiously, we observed that ubiquitination of STAT2 was inhibited in cells infected with species B HAdVs, which correlated with higher levels of STAT2 protein in these cells (Fig. 2F). In addition to preventing transcriptional activation of ISGs more efficiently, species B viruses were more efficient at preventing nuclear translocation of RuvBL1/2 (Fig. 4).

The indifference of species B adenoviruses to IFN-α treatment was unexpected considering HAdV-C2 showed a nearly 80% reduction in titers when the infected cells were pre-treated with IFN-α (Fig. 1A) and previous reports showing some sensitivity of species B viruses to IFN-β and γ (26–28). Since IFNs represent the front-line defense against viral invaders (47), it is logical for viruses to target this pathway early in the infectious process. Indeed, HAdV-C5 expresses the VAI and VAII protein kinase R (a major IFN effector molecule) inhibitory RNAs within 3 hours of infection (48). Our results demonstrate that the more pathogenic species B viruses have evolved effective strategies for blocking type I IFN and preventing the ill effects of IFN on viral replication. IFN is used as a treatment for viral infection (49), as cellular exposure to IFN will often reduce viral replication and enable quicker recovery. The observation that species B HAdVs appear resistant to type I IFNs suggests that their associated high levels of pathogenicity may be partly due to this resistance. Interestingly, when we investigated the effects of IFN on the plaque phenotype of the viruses tested (Fig. S6), we observed a large plaque phenotype with HAdV-B14 only. Further investigation revealed that this is likely due to premature apoptosis of the infected cells (data not shown) without affecting virus growth (Fig. 1A). Enhanced tissue damage due to cell death may lead to worse disease progression and outcomes as this would result in greater organ damage. Although this was an unexpected finding in our research, it suggests that the use of IFNs as therapeutics may need careful consideration depending on the type of pathogen that is being treated.

One potential mechanism of suppression of ISG activation that we observed to be more pronounced in species B HAdVs was reduced recruitment of STAT2 and RNA polymerase II to ISG promoters (Fig. 2E). We observed that the interaction of RuvBL1 with RNA polymerase II was inhibited with all viruses investigated, resulting in overall reduction of recruitment of RNA polymerase II to ISG promoters, as well as reduced recruitment of STAT2 to the same promoters. Although all viruses were able to significantly reduce the levels of STAT2 and RNA polymerase II at ISG promoters, species B viruses were particularly efficient at this as we did not detect significant levels of STAT2 or RNA polymerase II at ISG promoters after infection. An earlier study showed that STAT1 is sequestered in viral replication centers in HAdV-C5-infected cells (21). Although we did not see dramatic changes in STAT1 nor STAT2 sub-cellular localization (Fig. S10B), a close examination suggests that there may be more nuclear STATs in infected cells, with little to no difference between species C and species B viruses. The observed reduced ubiquitination of STAT2 may also play a role in ISG inhibition by species B HAdVs. Unexpectedly, this reduced ubiquitination correlated with higher STAT2 levels but reduced promoter recruitment *via* an unknown mechanism, which clearly merits further investigation.

Previously, it was shown that RuvBL1 is required for recruitment of RNA polymerase II to ISG promoters, whereas STAT2 was not (33). Although our studies show that RuvBL1 was essential for the suppression of ISGs by HAdV-C2, consistent with our previous work (23), it was not for HAdV-B7 and B14. Instead, it appears that RuvBL2 is more important for species B viruses than RuvBL1 as we observed impaired suppression of some ISGs when RuvBL2 was knocked down via siRNA. It is still not clear how species B viruses accomplish this. Our studies indicate that in infected cells, RuvBL1 forms high molecular weight complexes particularly when cells are infected with HAdV-B7 and B14, less so with HAdV-C2 (Fig. 2B). It is plausible that when these complexes are formed, RuvBL1 is sequestered away from ISG promoters and unable to bind. Indeed, we observed much more cytoplasmic RuvBL1 and RuvBL2 in cells infected with species B viruses, as well as more E1A that was also found in the cytoplasm (Fig. 4). An intriguing possibility is that E1A, RuvBL1, and RuvBL2 form a stable complex that may contain other proteins that is selectively retained in the cytoplasm and prevented from accessing ISGs. We and others have previously observed a similar strategy for sub-cellular redistribution by HAdV with other cellular proteins, such as Nek9 (46, 50), Ku70 (45), DREF (51), FUBP1 (44), and PKA (52).

The role of DCAF7 and FoxK, previously shown to play a role in ISG suppression by E1A (22), shows minimal impact on ISG suppression by species B HAdVs. Sequence comparisons of the binding regions for DCAF7 and FoxK (Fig. 1C) show strong conservation in residues binding to DCAF7, but weaker conservation for FoxK binding site with the threonine residues at position 183 in E1A243R of HAdV-C2 replaced with a proline at the equivalent position in HAdV-B7 and B14. Interestingly, we observed relatively weak binding of species B E1As to DCAF7 and no binding to FoxK; the former is somewhat surprising considering the conservation of the binding site while we expected the latter since the binding site is missing in species B HAdVs (53, 54). Ultimately, the effects of DCAF7 and FoxK on ISG suppression by species B HAdV are modest and variable, changing between the virus and the ISG with effects of FoxK knockdown possibly an indirect result of its role in STAT1/2 activation (55) rather than direct influence by HAdV. It is also unclear how chromatin is modified differentially between the different species. Previous studies have implicated the histone variant H2A.Z as a negative regulator of ISG activation (40) mediated by the acetyltransferase GCN5. We only observed small differences in H2A.Z loading at ISG promoters following virus infection (Fig. S8), similar to the effects observed on the constitutively active *ACTB* promoter. Knockdown of GCN5 had no significant effect on ISG activation after infection, while knockdown of H2A.Z slightly reduced ISG activation following infection, but this was not significant (Fig. S9). Collectively, although GCN5 is a known target of E1A from several different species of HAdV (56), it does not appear to be involved in ISG suppression by the serotypes we investigated. Similarly, H2A.Z, which co-localizes with trimethylated H3 lysine 3 (41), loading at ISG promoters was not specific and only showed general changes observed on a non-IFN-regulated promoter as well (Fig. S8). Collectively, although we observed a general increase in histone loading at cellular promoters, the exact chromatin dynamics of ISGs affected by HAdV-B7 and B14 remain to be fully elucidated.

To obtain a global picture of proteomic changes induced by the three different HAdV types, we performed shotgun proteomic analysis after IFN treatment and infection (Fig. 5; Fig. S12 to S16). This analysis reveals several significant differences between HAdV-C2, B7, and B14. Specifically, activation of a variety of energy-producing metabolic pathways by HAdV-B14 suggests that this virus is particularly good at driving and supporting its replication (Table 1), supported by its relatively rapid growth as compared to HAdV-C2 and B7 (Fig. 1A). Surprisingly, this did not occur with HAdV-B7, which, instead, was particularly efficient at suppressing a variety of innate immune pathways, such as MDA5 (57) and IFN (Table 2). These observations suggest significant differences in how species B-associated pathogenicity may be induced. Specifically, both viruses are highly efficient at suppressing ISG activation. While species B14 is highly efficient in driving metabolic pathways and inhibiting immune-mediated cell death, which may support a rapid, prolonged, and highly productive replicative cycle. Whereas species B7 is highly efficient at suppressing additional innate immune pathways, and T cell-mediated cell death pathways, which may enable this virus to evade immunity longer, driving greater pathogenicity.

In conclusion, our study represents a pioneering investigation providing molecular insights into how the highly pathogenic strains of HAdV, specifically B7 and B14, differ in their sensitivity to IFN and suppression of the IFN signaling pathway from species C viruses, and how they affect the host cell proteome. Our results show that species B HAdVs are not affected by IFN treatment in A549 cells and they are much more efficient at ISG suppression after infection. Importantly, we show that these viruses are very efficient at preventing the recruitment of STAT2 and RNA polymerase II to ISG promoters in cells treated with IFN. Significantly, this work highlights the dramatic differences in how different HAdV species can affect ISG activation and cellular response to IFN. Future work will further unravel the molecular mechanisms of how these viruses can efficiently take over the cell. Significantly, our current work focused on the viral response to IFN-α only and the role of E1A in blocking this IFN response. Much remains to be answered regarding the effects of other IFNs on viral replication and the impact of HAdV genes from species B viruses on IFN suppression, with many questions remaining about the functions of the early regions E1, E3, and E4.

## MATERIALS AND METHODS

### Antibodies

Mouse monoclonal anti-species C E1A M73 and M58 antibodies were previously described in reference (58), mouse monoclonal anti-hexon 27F11 and 9C12 antibodies in reference (59), and mouse monoclonal anti-hemagglutinin (anti-HA clone 12CA5) in (60); all were grown in-house and used as hybridoma supernatant. Anti-histone H3 and anti-DCAF7 antibodies were purchased from Abcam (cast. ab180727 and ab138490, respectively), while anti-tri-methyl K4 H3 and anti-H2A.Z antibodies were purchased from Cell Signaling Technology (cats. C42D8 and E9M5G, respectively). Rabbit anti-RuvBL1 and RuvBL2 antibodies were ordered from Cell Signaling Technology (cat. D1L8J) and Abclonal (cat. A1905) respectively; and anti-STAT1, anti-STAT2 and anti-FoxK1 and anti-FoxK2 antibodies were ordered from Cell Signaling Technology (cats. D1K9Y, D9J7L, 12025, and 12008 respectively). Anti-RNA polymerase II antibody (CTD specific) and anti-adenovirus structural protein antibody were ordered from Abcam (cats. Ab26721 and Ab6982 respectively). Rat anti-HA antibody (Roche, cat. 11867423001), clone 3F10, was used for western blot. Secondary antibodies were purchased from Jackson ImmunoResearch.

Rabbit polyclonal antibodies for HAdV-B7 and B14 E1A and DBP proteins were newly generated by Pacific Immunology using the following peptide sequences:

Ad7/14 E1A—KLEDLLEGGDGPLDLSTRK

Ad7/14 DBP—QFRNVSLPAGHYDSRQNPFD

These antibodies were affinity purified using peptide columns before use. Note that for E1A detection and other viral proteins, the variable levels observed between the different viruses are not necessarily due to differences in viral protein levels but are rather due to the fact that different antibodies had to be used to detect different viral species or are caused by variability in antibody sensitivity for different viral species.

### Cell and virus culture

A549 (ATCC# CCL-185) and HT1080 (ATCC# CCL-121) cells were grown in Dulbecco’s Modified Eagle’s Medium (MilliporeSigma) supplemented with 5% fetal bovine serum, streptomycin, and penicillin (Corning). IMR-90 cells (ATCC# CCL-186) were grown in DMEM supplemented with 10% fetal bovine serum with streptomycin and penicillin. Unless otherwise specified, all virus infections were carried out at an MOI of 10 in serum-free media for 1 hour after which fresh complete media was added without removal of the infection media. For most experiments, A549 cells were used; however, in cases where transfection was necessary we used HT1080 cells due to their favorable transfection properties.

### Chromatin immunoprecipitation

ChIP was carried out essentially as previously described (29, 31). Cells were infected with the indicated adenoviruses at a MOI of 10 and harvested 24 hours after infection for ChIP analysis. IFN α-2A treatment (final concentration 1 U/mL) was performed when specified in figure legends.

qPCRs were carried out for ISG promoters using PowerUp SYBR Green Master Mix (BioRad) according to the manufacturer’s directions using a BioRad CFX96 Real-Time System (BioRad). The annealing temperature used was 60°C, and 40 cycles were run.

### Cross-linked immunoprecipitation

Fifteen-centimeter plates of A549 cells transfected to express HA-RuvBL1 24 hours earlier were infected with HAdV-C2, B7, or B14 for 1 hour at MOI 10. 16 hours later, IFN α-2A was applied (final concentration 1 U/mL). Eight hours later (24 hours after initial infection), cells were cross-linked directly on the plate with 1% (final concentration) formaldehyde for 10 minutes. Glycine was then applied to a final concentration of 125 mM to stop the cross-linking reaction. Cells were scraped and pelleted, and the pellets were washed with PBS three times. These pellets were lysed with NP-40 lysis buffer [0.5% NP-40, 50 mM Tris (pH 7.8), 150 mM NaCl], and immunoprecipitation was performed using 12CA5 anti-HA antibody. Samples were resolved on Invitrogen Bolt 4-12% Bis-Tris Plus gels in MOPS buffer (Invitrogen).

### Cytopathic effect imaging

Six-well plates of A549 or IMR-90 cells, pretreated with IFN α-2A by 16 hours where indicated, were infected with HAdV-C2, B7, and B14 at an MOI indicated in the specific figure legend, as described above. Images were taken *via* BioRad ZOE Fluorescent Cell Imager in brightfield at the indicated timepoints.

### Cytoplasmic/nuclear fractionation

A549 cells were plated on 10 cm plates and infected with HAdV-C2, B7, and B14 at MOI 100. IFN was applied 16 hours after infection, and cells were harvested 8 h after that. Cells were pelleted and washed with cold PBS twice before being resuspended in hypotonic salt solution [20 mM Tris (pH 7.4), 10 mM NaCl, 3 mM MgCl_2_] and left on ice for 15 minutes. NP-40 detergent was added to 0.5% final concentration and cells were lysed *via* vortex. Nuclei were pelleted and the supernatant was isolated as the cytoplasmic fraction before nuclei were lysed in cell extraction buffer [10 mM Tris (pH 7.4), 100 mM NaCl, 1% Triton X-100, 1 mM EDTA, 10% glycerol, 0.1% SDS, 0.5% deoxycholate] supplemented with protease inhibitor cocktail. The remaining cell debris was pelleted and the supernatant was isolated as the nuclear fraction. Protein levels were quantified via Bradford assay and resolved *via* SDS-PAGE.

### Immunofluorescence

A549 cells or HT-1080 cells, depending on the experiment, were plated at low density (~40,000 cells per chamber) on chamber slides (Nalgene Nunc) and subsequently infected as described. Twenty-four hours after infection, cells were fixed in 4% formaldehyde, permeabilized in 0.1% Triton, blocked in blocking buffer (1% normal goat serum, 1% BSA, 0.2% Tween-20 in PBS), and stained with specific primary antibodies. M58, M73, 27F11, and 9C12 were used neat (hybridoma supernatant); RuvBL1, RuvBL2, STAT1, and STAT2 antibodies were used at a dilution of 1:400, and AlexaFluor-488 and -594 secondary antibodies (Jackson ImmunoResearch) were used at a dilution of 1:600. After staining and extensive washing, slides were mounted using Prolong Gold with DAPI (Invitrogen) and imaged using Zeiss LSM700 confocal laser scanning microscope. Images were analyzed using the Zeiss ZEN software package.

### Immunoprecipitation

A549 cells were lysed in NP-40 lysis buffer supplemented with a protease inhibitor cocktail (MilliporeSigma). The cell lysate was used for IP with the rabbit anti-RuvBL1, mouse anti-HA 12CA5, mouse anti-E1A (species C), or rabbit anti-E1A (species B) antibody depending on the specific experiment. For ubiquitination IPs RIPA buffer [150 mM NaCl, 1% NP-40, 0.5% deoxycholate, 0.1% SDS, and 50 mM Tris pH (7.4)] supplemented with 25 mM N-ethylmaleimide (MilliporeSigma) was used instead of the NP-40 lysis buffer.

### Interferon overlay assay

Six-well plates of A549 cells were infected with serial dilutions of HAdV-C2, B7, and B14. After infection, infection media was removed and 3 mL of 1-part 2× DMEM and 1-part agarose was applied to each well. Where indicated, the agarose-DMEM overlay contained IFN α-2A at a final concentration of 1 U/mL. Plates were incubated at 37°C, 5% CO_2_ until plaques began to show. Wells were then topped off with 2 mL of 0.01% Neutral Red (Fisher cat. 553–24-2)-containing agarose-DMEM and incubated for 24 hours. Plaques were imaged *via* AlphaEaseFC software in a FluorChem 8900 (Alpha Innotech) gel imaging cabinet. Images were enhanced with a 3D effect for ease of plaque visualization.

### PCR primers

qPCR primers for adenovirus species C viral genes and GAPDH were previously described in (45). Those for IFI6, IFI6 promoter, and IFIT1 promoter are previously described in reference (23). All remaining primers (additional ISGs and promoters, all species B viral genes) are described in Fig. S17. All primers were generated through the services of Integrated DNA Technologies.

### Plasmids

The expression plasmid for HA-RuvBL1 was generated by PCR-amplifying the genetic sequence of RuvBL1 and cloning it into the pcDNA-HA expression vector. The HA-RuvBL2 expression vector was obtained from Addgene (plasmid #51636) (61).

### Proteomic analysis

A549 cells were infected with the different HAdV strains at MOI 10 for 16 hours and then treated with IFN. After 8 hours, cells were harvested and lysed in PBS using sonication using a Covaris M220 focused ultrasonicator, and total proteins were precipitated using trichloroacetic acid prior to analysis. Protein samples were sent to the Southern Alberta Mass Spectrometry Centre for 2D-LC-MS/MS shotgun proteomics analysis, where raw reads were interpreted into Scaffold data. Results were analyzed broadly by Heatmapper software (62), and in detail by Cytoscape’s (63) String function and clusterMaker app (64).

### Real-time gene expression analysis

Cells were infected with indicated viral strains at an MOI of 10 for 24 hours with IFN α-2A applied to a final concentration of 1 U/mL 8 hours prior to harvest. Total RNA was extracted using TRIzol Reagent (MilliporeSigma) according to the manufacturer’s instructions. 1 µg of total RNA was used for cDNA generation with SuperScript VILO reverse transcriptase (Invitrogen) according to the manufacturer’s guidelines, using random hexanucleotides for priming. The cDNA was subsequently used for real-time expression analysis *via* BioRad CFX96 real-time thermocycler. Analysis of expression data was carried out and was normalized to *glyceraldehyde 3-phosphate dehydrogenase* (*GAPDH*) mRNA levels. Total E1A mRNA was detected as previously described (65).

### SiRNA knockdowns

Knockdowns were performed *via* siLentFect Lipid Reagent for RNAi (BioRad) according to the manufacturer’s specifications. Knockdown conditions were maintained for 24 hours for H2A.Z, and 72 hours for RuvBL1, RuvBL2, and GCN5. Infections were then carried out as described with IFN α-2A application taking place 8 hours prior to harvest. Gene expression analysis was then carried out as described.

siRNAs for RuvBL1, RuvBL2, GCN5, and H2A.Z were obtained from Life Technologies (siRNA IDs s16369, s21307, s5657, and s6414 respectively).

### Statistical analysis

Statistical analysis for all experiments was performed using a two-tailed Student’s *t*-test using HAdV-C2-infected samples as the reference for comparison. *P*-values of ≤0.05 were considered statistically significant. Statistical significance is indicated with asterisks when present.

### Transfections

Media was changed on cells 20 minutes prior to transfection. Transfections were prepared by mixing 1 mL of serum-free DMEM, 10 µg of total plasmid DNA, and 20 µL of 1 mg/mL solution of linear polyethylenimine 25 kDa reagent from Polysciences (cat. 23966–2). This mixture was vortexed for 10 seconds and incubated at room temperature for 20 minutes. The transfection complexes were then added to the cells and incubated for 24 to 48 hours. Note that reagent quantities assume application to a 10 cm plate of cells (fill volume 10 mL). For other plate/well sizes, reagent quantities were altered proportionally to the fill volume.

### Viruses

Viruses used in the study were HAdV-C2 (ATCC# VR-846), HAdV-B7 (ATCC# VR-7), and HAdV-B14 (ATCC# VR-15). All infections were carried out in serum-free medium for 1 hour at an MOI of 10 unless otherwise specified in figure legends.

### Virus growth assay

A549 cells were infected with HAdV-C2, B7, or B14 at MOI 10 in serum-free media, 16 hours after the cells were treated with IFN α-2A (final concentration 1 U/mL). The virus was adsorbed for 1 hour at 37°C and 5% CO_2_. Virus titers were determined 24, 48, and 72 hours after infection by freeze-thaw and plaque assays performed on A549 cells by serial dilution.

### Western blot

All protein samples were boiled in DTT-enriched protein loading dye at 100°C for 10 minutes. Samples were resolved on Invitrogen Bolt 4%–12% Bis-Tris Plus gel in MOPS buffer or MES buffer (both from Invitrogen) depending on the size of the target protein. Gels were transferred to the PVDF membrane *via* Genscript eBlot L1 blot transfer apparatus. Membranes were blocked for 1 hour in skim milk powder in TBST. Primary antibodies were applied in 3% BSA, shaking, overnight. Secondary antibodies were applied in a blocking buffer, diluted 1:100,000.

## Data Availability

All proteomics data have been deposited in the PRIDE database under accession number PXD045673, DOI: 10.6019/PXD045673.

## SUPPLEMENTAL MATERIAL

Figure S1 mbio.01038-24-s0001.tif IFN effect on HAdV cytopathic effect.

Figure S2 mbio.01038-24-s0002.tif Previously observed patterns are maintained in primary cells.

Figures S3 and S4 mbio.01038-24-s0003.pdf IFN effect on viral gene and protein expression.

Figures S5 to S7 mbio.01038-24-s0004.pdf IFN effect on HAdV entry, large-plaque phenotype, and RuvBL1 recruitment.

Figures S8 and S9 mbio.01038-24-s0005.pdf HAdV in histone recruitment and HAdV ISG suppression.

Figure S10 mbio.01038-24-s0006.png RuvBL1 and RuvBL2 localization.

Figure S11 mbio.01038-24-s0007.pdf HAdV infection and STAT2-RNA polymerase 2 interaction.

Figure S12 mbio.01038-24-s0008.tif Clusters of proteins upregulated in HAdV-infected cells.

Figure S13 mbio.01038-24-s0009.tif Clusters of proteins downregulated in HAdV-infected cells.

Figure S14 to S17 mbio.01038-24-s0010.pdf Clusters of proteins up- and downregulated in HAdV-B-infected cells, heat map of proteomic data, and primers.

ASM does not own the copyrights to Supplemental Material that may be linked to, or accessed through, an article. The authors have granted ASM a non-exclusive, world-wide license to publish the Supplemental Material files. Please contact the corresponding author directly for reuse.
