# Supplementary figures and images for: Molecular insights into type I interferon suppression and enhanced pathogenicity by species B human adenoviruses B7 and B14
Source: mBio. 2024 Jun 28;15(8):e01038-24. doi: 10.1128/mbio.01038-24 (PMC11323573; doi:10.1128/mbio.01038-24)

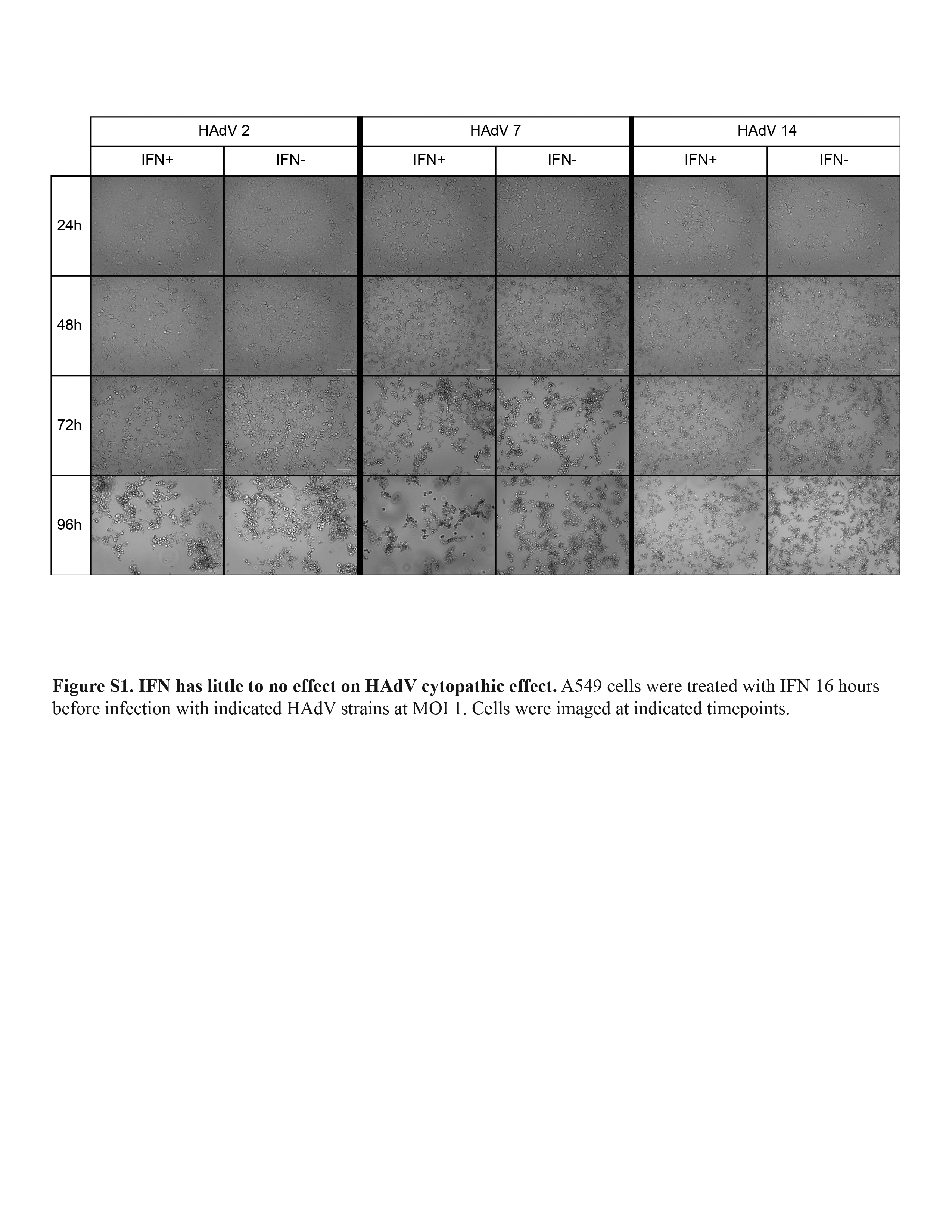

Supplement: Figure S1 — IFN effect on HAdV cytopathic effect. [file mbio.01038-24-s0001.tif]

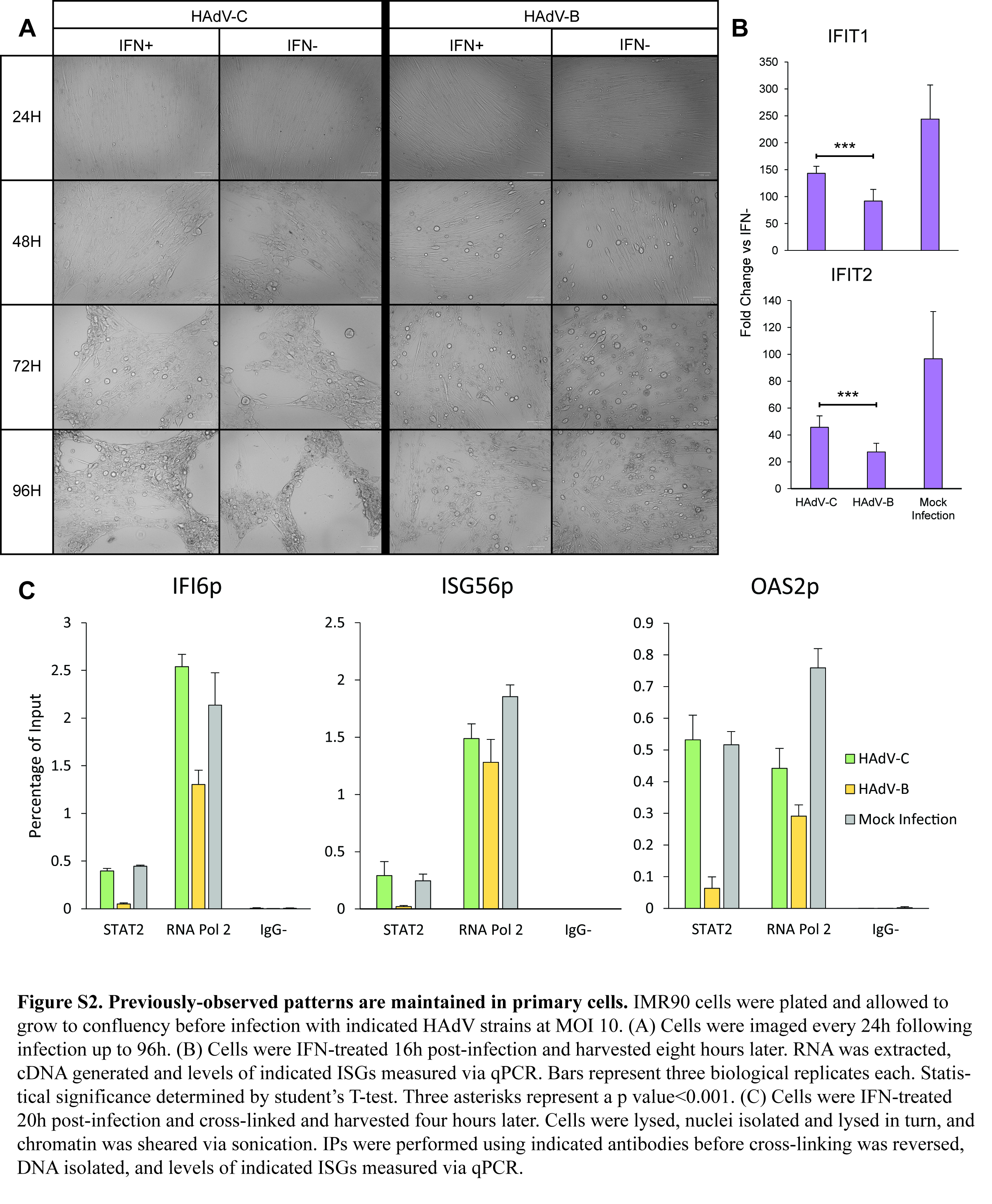

Supplement: Figure S2 — Previously observed patterns are maintained in primary cells. [file mbio.01038-24-s0002.tif]

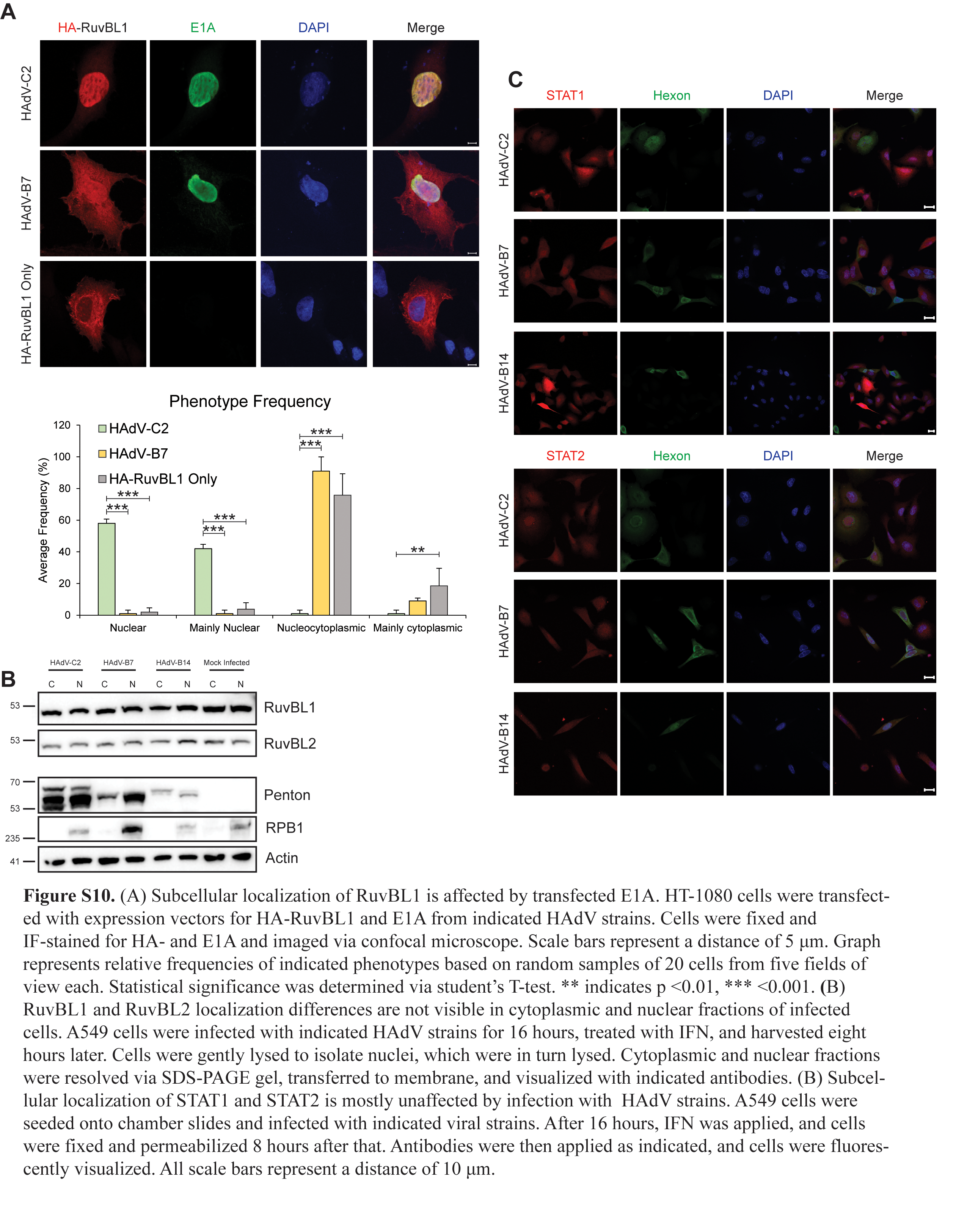

Supplement: Figure S10 — RuvBL1 and RuvBL2 localization. [file mbio.01038-24-s0006.png]

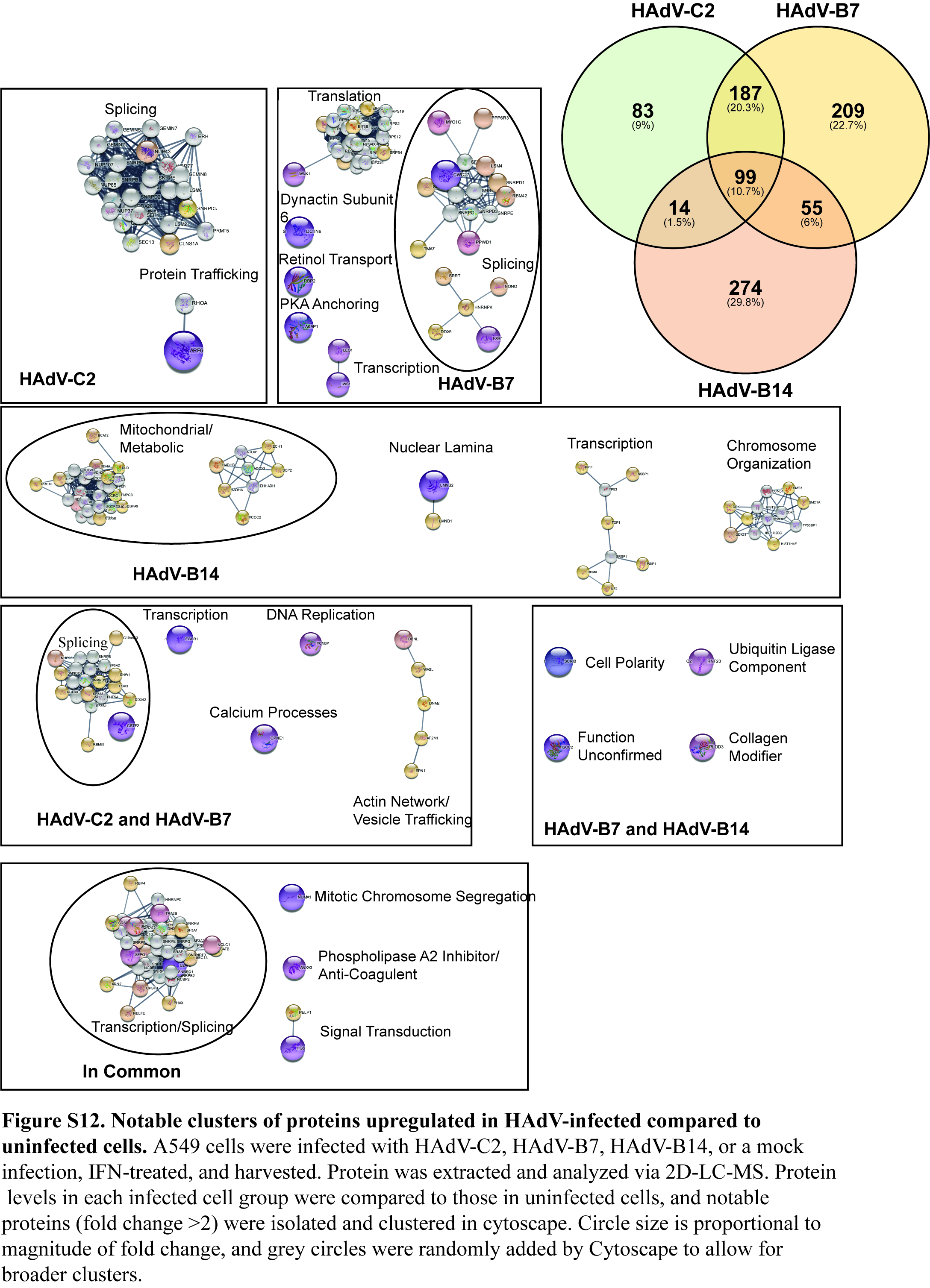

Supplement: Figure S12 — Clusters of proteins upregulated in HAdV-infected cells. [file mbio.01038-24-s0008.tif]

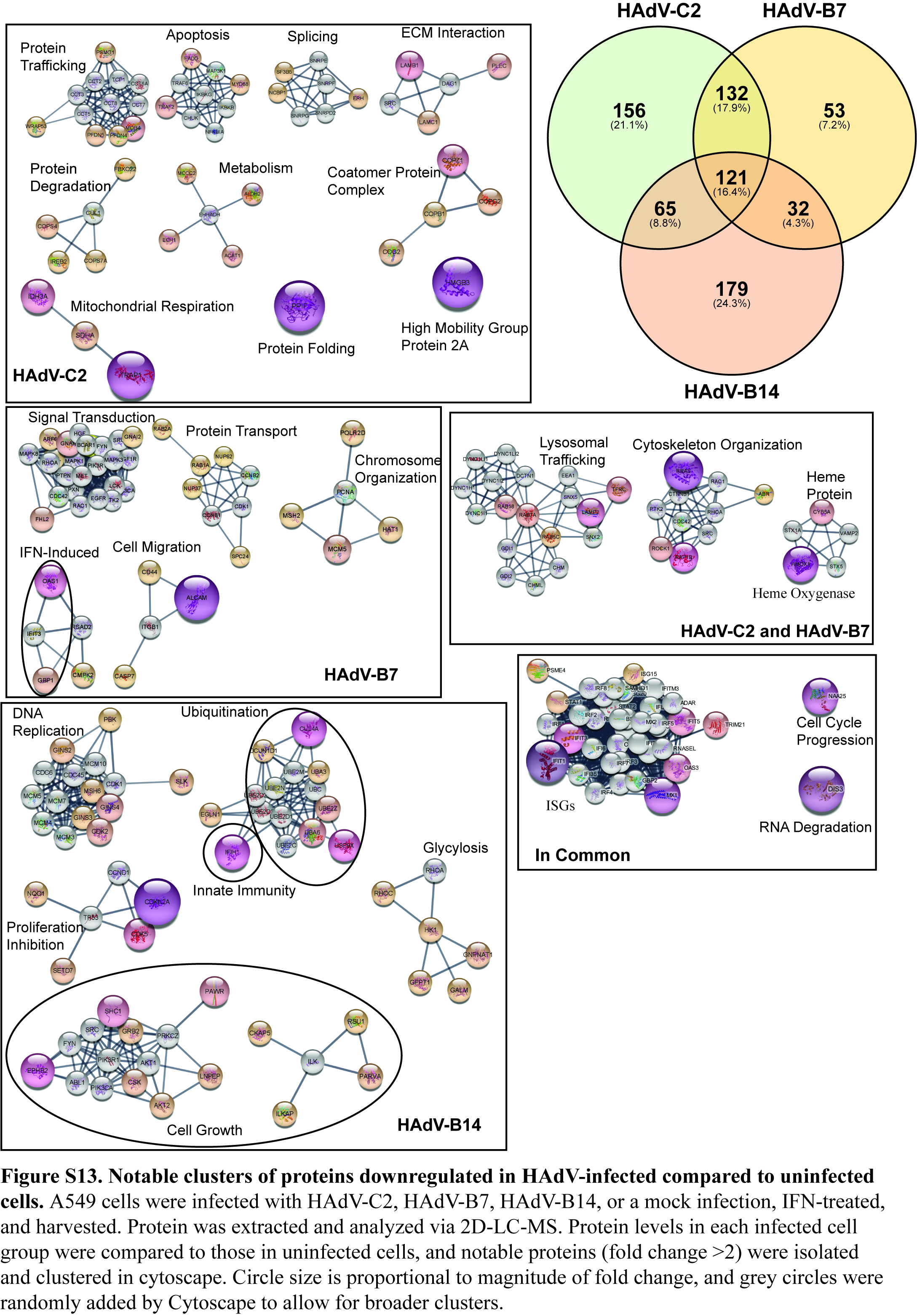

Supplement: Figure S13 — Clusters of proteins downregulated in HAdV-infected cells. [file mbio.01038-24-s0009.tif]
