# Supplementary material for: Molecular insights into type I interferon suppression and enhanced pathogenicity by species B human adenoviruses B7 and B14
Source: mBio. 2024 Jun 28;15(8):e01038-24. doi: 10.1128/mbio.01038-24 (PMC11323573; doi:10.1128/mbio.01038-24)
Supplement: Figures S3 and S4 — IFN effect on viral gene and protein expression. [file mbio.01038-24-s0003.pdf]

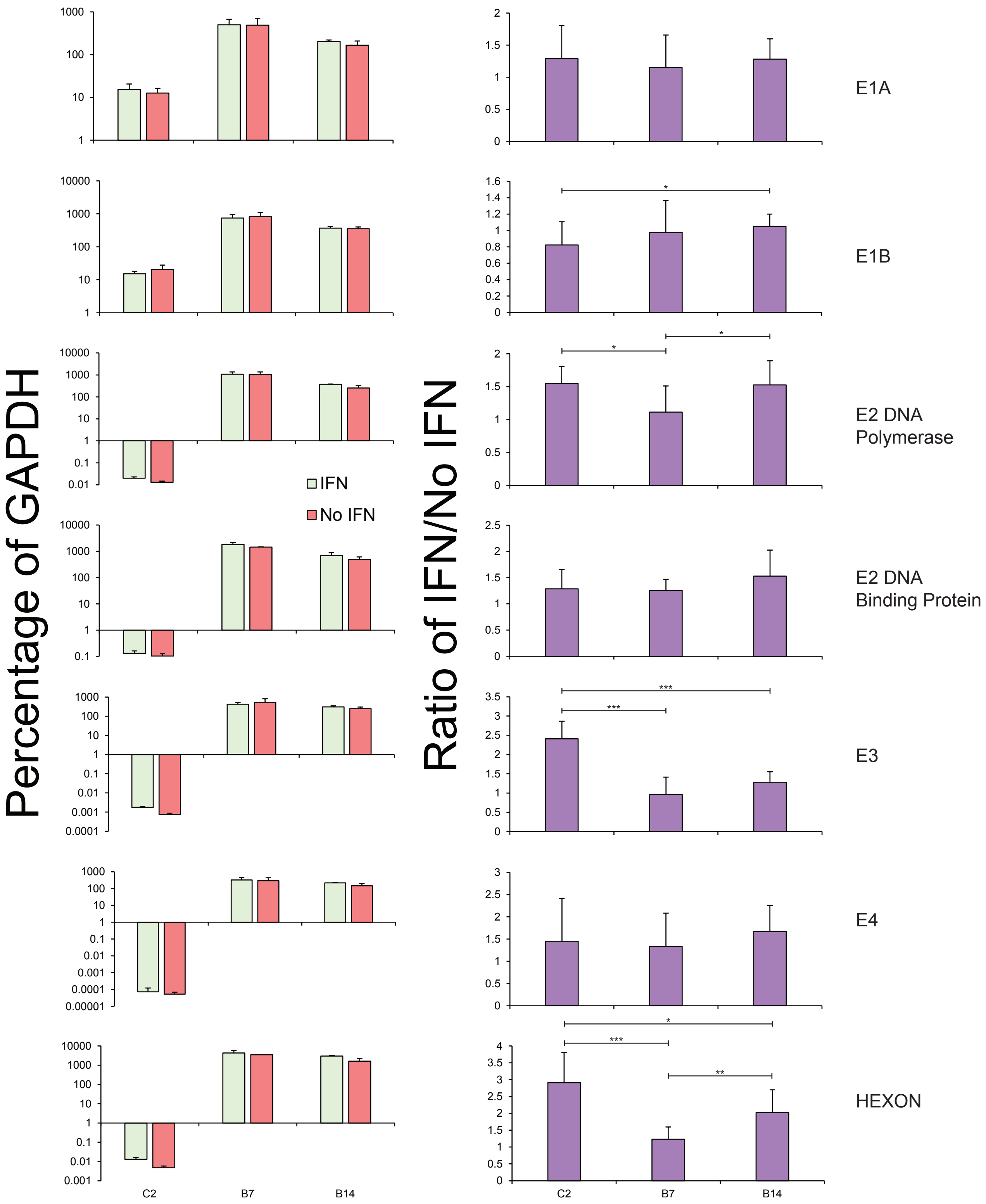

Figure S3. Viral gene expression is not broadly affected by the presence of IFN. A549 cells were treated with IFN α-2A 16 hours after infection with indicated HAdV strains. Eight hours later, the cells were harvested, RNA extracted, and cDNA generated. Viral gene expression was analyzed via real-time qPCR. Results were normalized to percentage expression of housekeeping gene GAPDH and presented as-is (A) and as a ratio of IFN-treated over untreated (B). All experiments were performed in triplicate. Statistical analysis was performed via unpaired t-test. * signifies a p-value of <0.05, ** represents <0.01, and *** indicates <0.001.

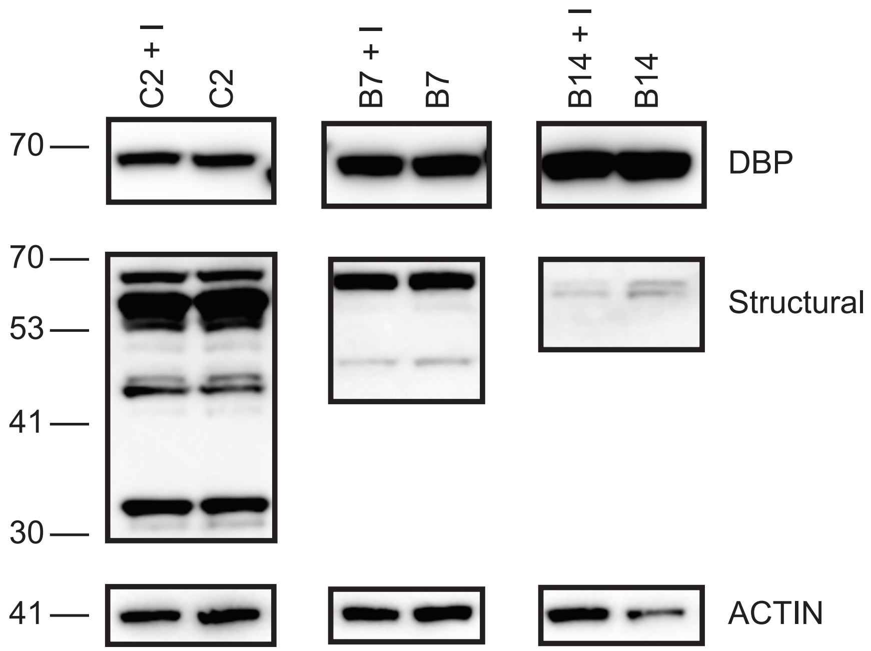

Figure S4. Viral protein expression levels are unaffected by the presence of interferon. A549 cells were infected with infected with indicated HAdV strains, treated with IFN 16 hours later, and harvested eight hours after that. Cells were lysed, protein levels measured, and equal protein volumes resolved on SDS gel, transferred to PVDF membrane, and blotted with indicated antibodies.
