# Supplementary material for: Molecular insights into type I interferon suppression and enhanced pathogenicity by species B human adenoviruses B7 and B14
Source: mBio. 2024 Jun 28;15(8):e01038-24. doi: 10.1128/mbio.01038-24 (PMC11323573; doi:10.1128/mbio.01038-24)
Supplement: Figures S5 to S7 — IFN effect on HAdV entry, large-plaque phenotype, and RuvBL1 recruitment. [file mbio.01038-24-s0004.pdf]

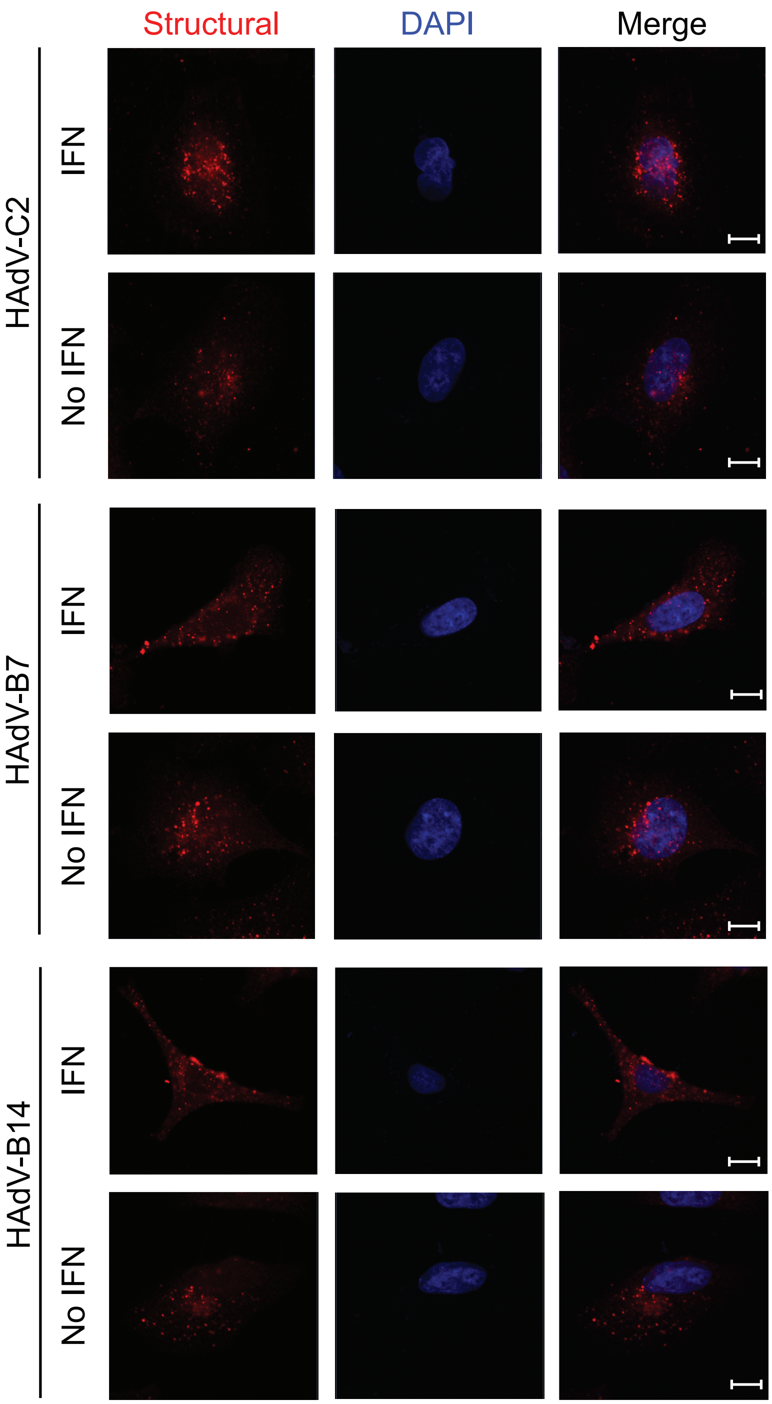

Figure S5. HAdV entry is unaffected by the presence of IFN. A549 cells were treated with IFN 16 hours prior to infection with indicated HAdV strains. Two hours after infection, cells were fixed, permeabilized, and stained with indicated antibodies. Cells were imaged by immunofluorescent microscopy. Scale bars represent a distance of 5 μm.

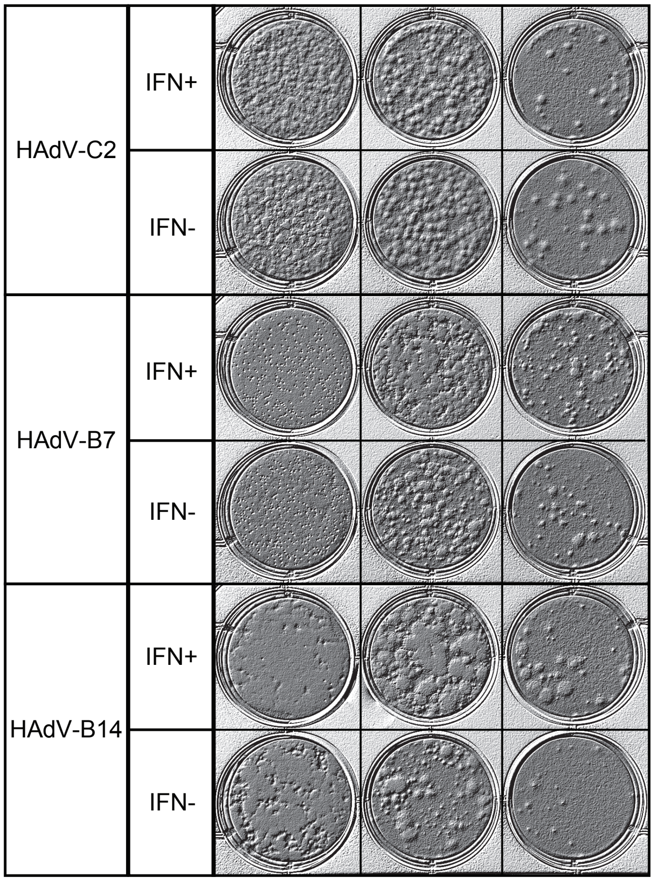

Figure S6. Presence of IFN generates a distinct large-plaque phenotype in HAdV-B14-infected cells. A549 cells were infected with serial dilutions of indicated HAdV strains and overlayed with agar-DMEM containing IFN where indicated. Once plaques had appeared, overall well appearance was visualized by neutral red dye.

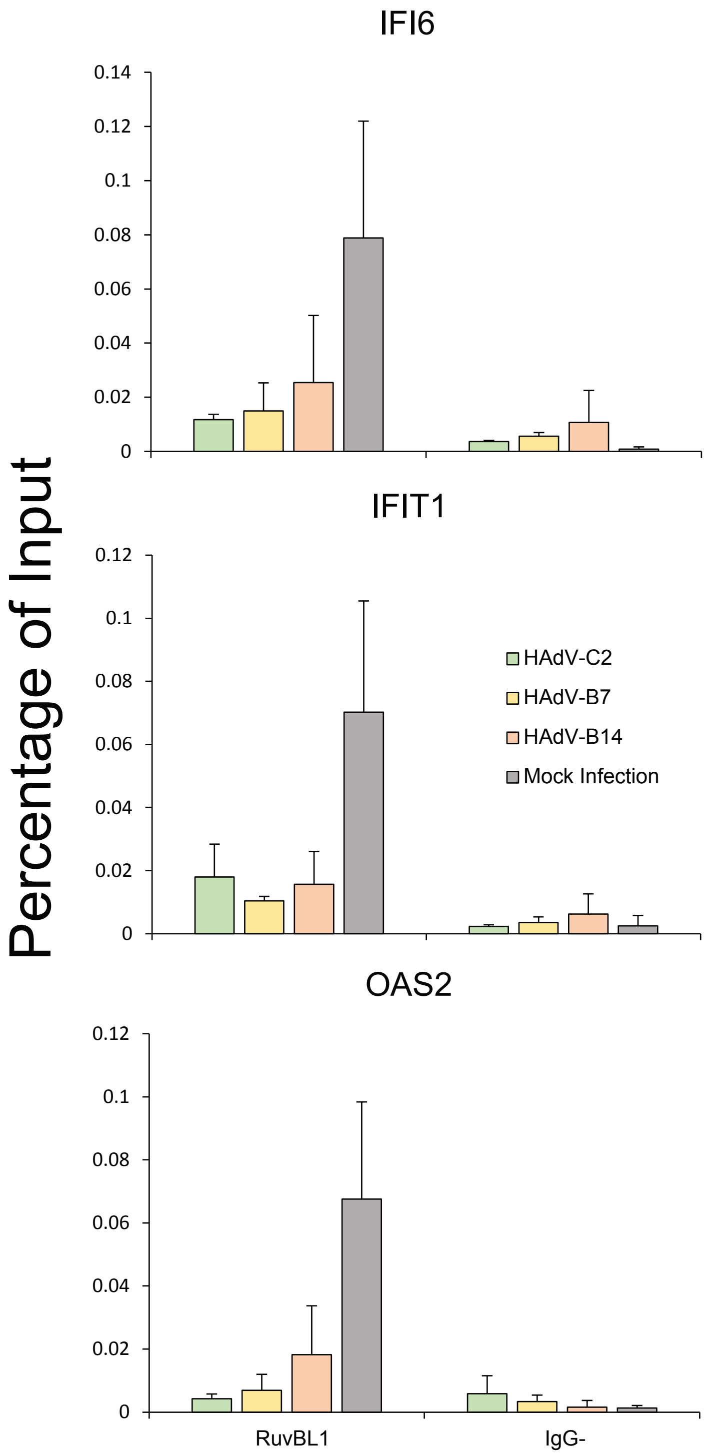

Figure S7. Recruitment of RuvBL1 to ISG promoters in IFN-treated cells is universally reduced during HAdV-infection. A549 cells were infected with indicated HAdV strains for 24 hours, treated with IFN, and harvested eight hours later. Cells were cross-linked and lysed, and RuvBL1 was immunoprecipitated. Cross-linking was reversed, DNA isolated, and relative ISG promoter levels analyzed via qPCR. Results based on biological duplicate samples. Statistical significance determined by student's T-test.
