# Supplementary material for: Molecular insights into type I interferon suppression and enhanced pathogenicity by species B human adenoviruses B7 and B14
Source: mBio. 2024 Jun 28;15(8):e01038-24. doi: 10.1128/mbio.01038-24 (PMC11323573; doi:10.1128/mbio.01038-24)
Supplement: Figures S8 and S9 — HAdV in histone recruitment and HAdV ISG suppression. [file mbio.01038-24-s0005.pdf]

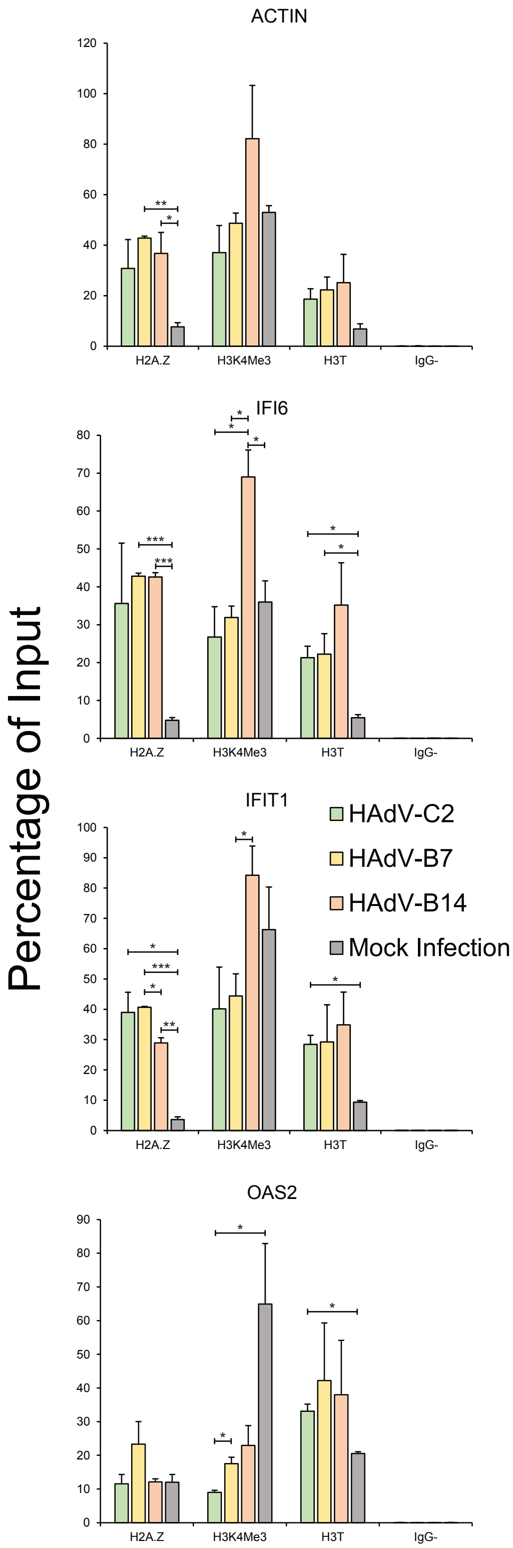

Figure S8. HAdV alters histone recruitment to ISG promoters as part of universal histone alteration. A549 cells were treated with interferon α-2a at a concentration of 1 unit/mL 20 hours before infection with indicated HAdV stocks. 24 hours after infection, cells were cross-linked and harvested, and indicated proteins were immunoprecipitated. Cross-linking was reversed, DNA isolated, and relative levels analyzed via qPCR. Bars represent biological duplicates. Statistical significance determined by student's T-test and indicated where present. * represents p<0.05, ** p<0.01, and *** p<0.001.

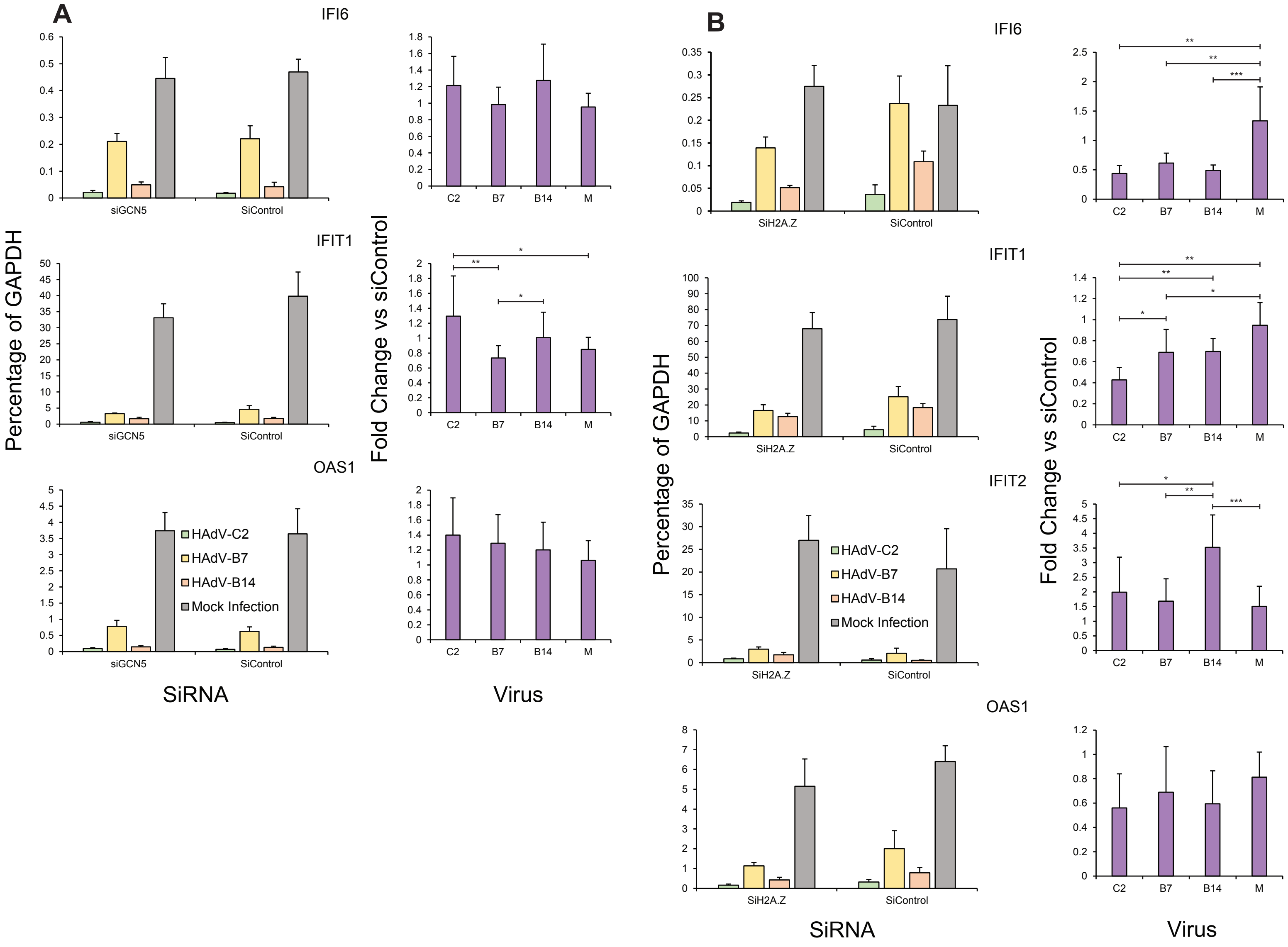

Figure S9. HAdV ISG suppression is mostly unaffected by knockdown of GCN5 or H2A.Z. A549 cells were treated with siRNA to knock down GCN5 (A) or H2A.Z (B) for 72 and 24 hours respectively. Cells were then infected with indicated HAdV strains and treated with IFN 16 hours later. After eight more hours, RNA was extracted, cDNA generated, and gene expression measured via qPCR. Results are presented as percentage of GAPDH (left column) and fold change versus cells treated with control siRNA (right column). Each column based on the results of biological triplicate samples. Statistical significance determined by student's T-test and noted where present. * represents p-value<0.05, ** represents p<0.01, *** represents p<0.001.
