# Supplementary material for: Molecular insights into type I interferon suppression and enhanced pathogenicity by species B human adenoviruses B7 and B14
Source: mBio. 2024 Jun 28;15(8):e01038-24. doi: 10.1128/mbio.01038-24 (PMC11323573; doi:10.1128/mbio.01038-24)
Supplement: Figure S11 — HAdV infection and STAT2-RNA polymerase 2 interaction. [file mbio.01038-24-s0007.pdf]

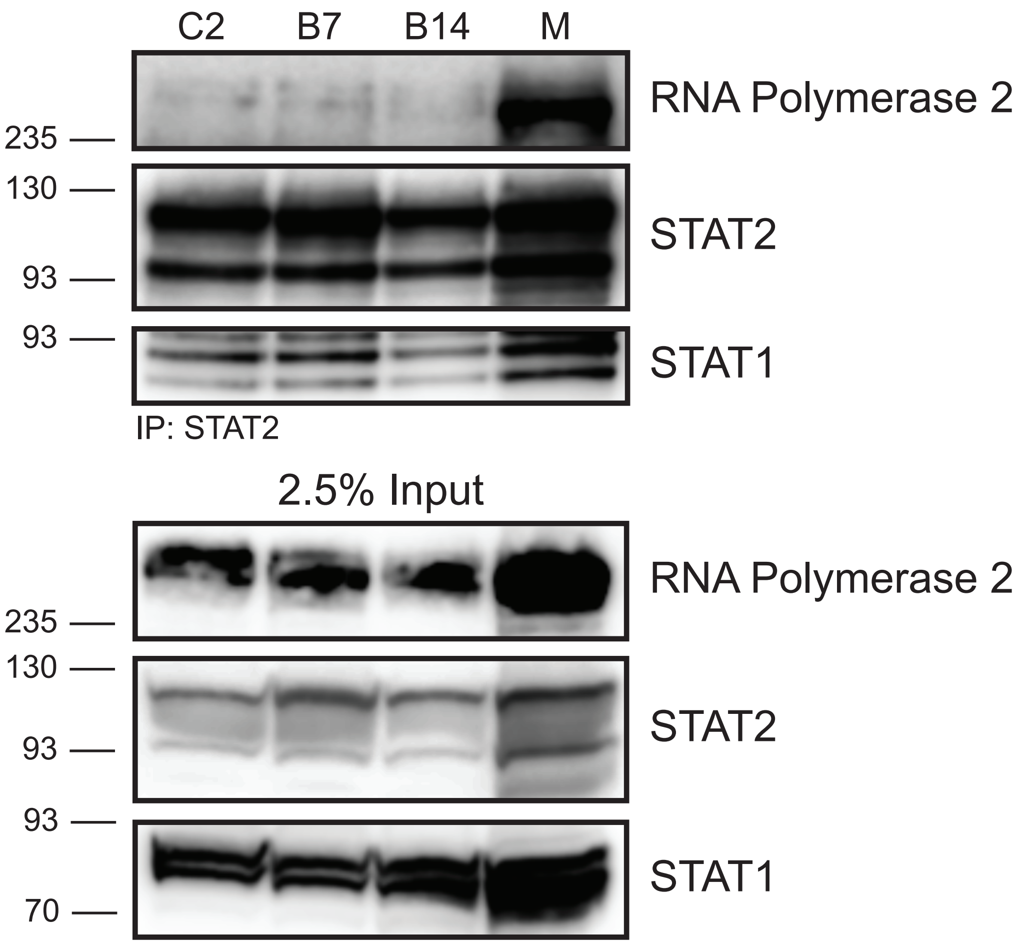

Figure S11. HAdV infection decreases STAT2-RNA Polymerase 2 interaction. A549 cells were treated with IFN and infected with indicated viral strains 16 hours later. Cell were harvested and lysed 72 hours after infection. Cell lysate was precipitated for STAT2, resolved on SDS gel, transferred to PVDF membrane, and blotted as indicated. M - mock infected, C2 - HAdV-C2, B7 - HAdV-B7, B14 - HAdV-B14.
