# Supplementary material for: Molecular insights into type I interferon suppression and enhanced pathogenicity by species B human adenoviruses B7 and B14
Source: mBio. 2024 Jun 28;15(8):e01038-24. doi: 10.1128/mbio.01038-24 (PMC11323573; doi:10.1128/mbio.01038-24)
Supplement: Figure S14 to S17 — Clusters of proteins up- and downregulated in HAdV-B-infected cells, heat map of proteomic data, and primers. [file mbio.01038-24-s0010.pdf]

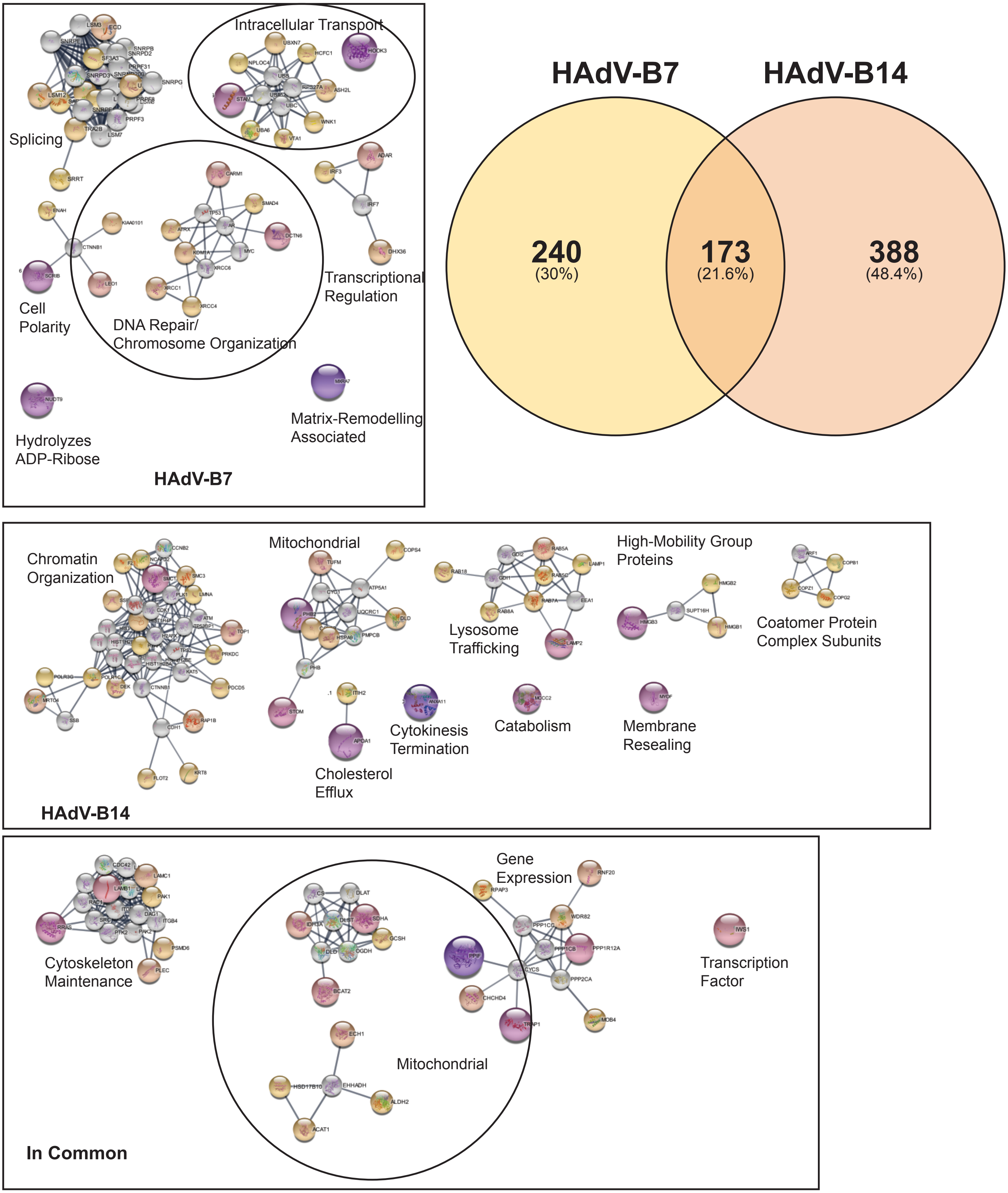

Figure S14 . Notable clusters of proteins upregulated in HAdV-B-infected compared to HAdV-C-infected cells. A549 cells were infected with HAdV-C2, HAdV-B7, HAdV-B14, or a mock infection, IFN-treated, and harvested. Protein was extracted and analyzed via 2D-LC-MS. Protein levels in each HAdV-B-infected cell group were compared to those in HAdV-C-infected cells, and notable proteins (fold change >2) were isolated and clustered in cytoscape. Circle size is proportional to magnitude of fold change, and grey circles were randomly added by Cytoscape to allow for broader clusters.

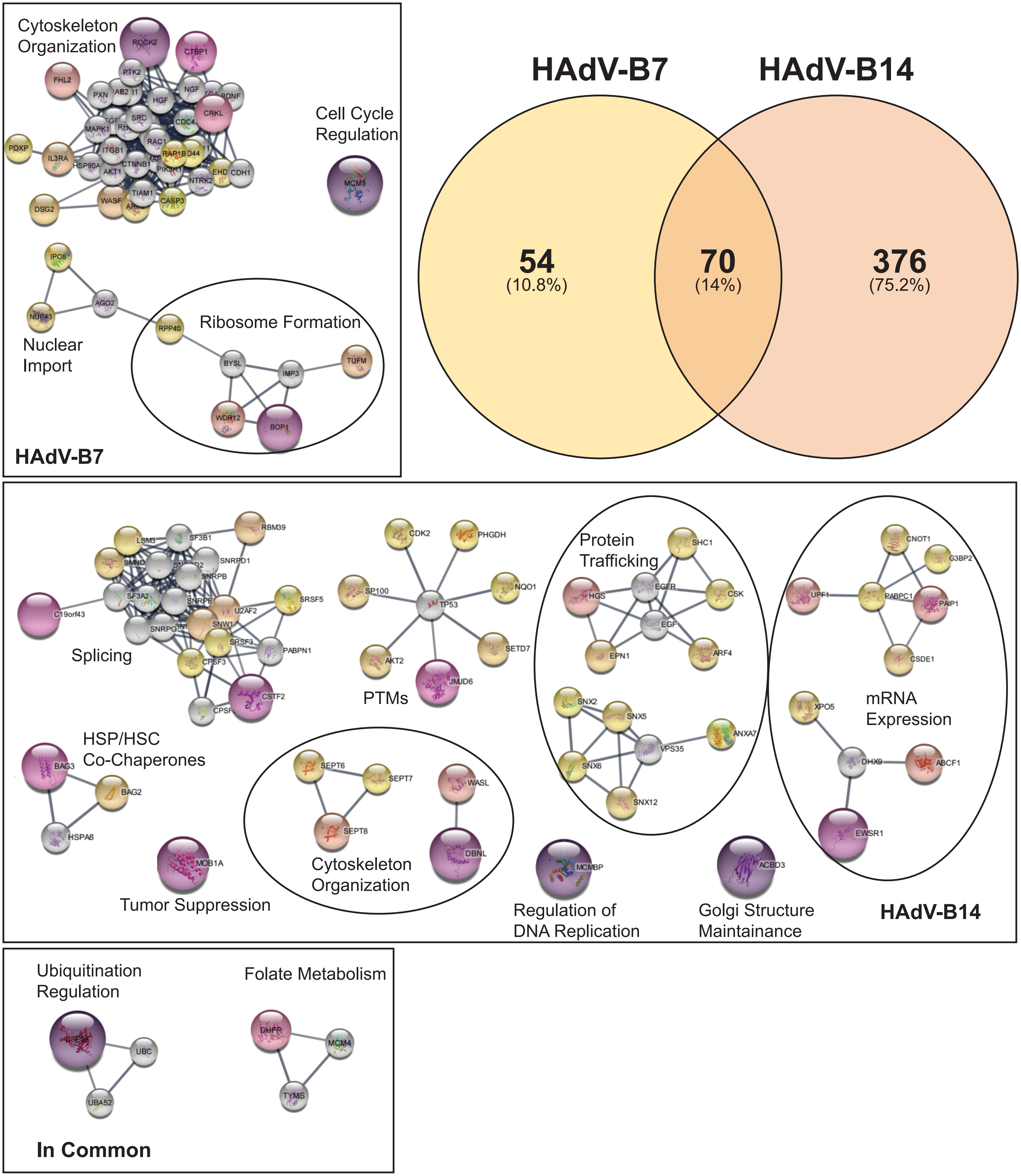

Figure S15. Notable clusters of proteins downregulated in HAdV-B-infected compared to HAdV-C-infected cells. A549 cells were infected with HAdV-C2, HAdV-B7, HAdV-B14, or a mock infection, IFN-treated, and harvested. Protein was extracted and analyzed via 2D-LC-MS. Protein levels in each HAdV-B-infected cell group were compared to those in HAdV-C-infected cells, and notable proteins (fold change >2) were isolated and clustered in cytoscape. Circle size is proportional to magnitude of fold change, and grey circles were randomly added by Cytoscape to allow for broader clusters.

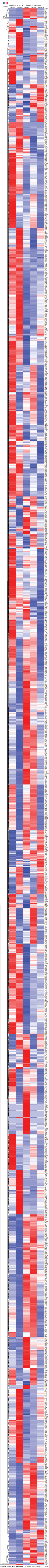

Figure S16. Heat map of total proteomic data. A549 cells were infected with HAdV-C2, HAdV-B7, HAdV-B14, or a mock infection, IFN-treated, and harvested. Protein was extracted and analyzed via 2D-LC-MS. Results were visualized via heat mapper software. Red indicates upregulation and blue represents downregulation.

	Name	Sequence
ISGs	IFIT1 F	AAAAGCCCACATTTGAGGTG
	IFIT1 R	GAAATTCCTGAAACCGACCA
	IFIT2 F	GCGTGAAGAAGGTGAAGAGG
	IFIT2 R	GCAGGTAGGCATTGTTTGGT
	OAS1 F	AGACCGACGATCCCAGGAGGT
	OAS1 R	TGGGGTGGATGCTGCCTGGA
	OAS2 F	GGGTGGAGGGGACCGTTGGT
	OAS2 R	CCTGGTGTCTGCATTGTCGGC
Promoters	OAS2p F	GCAAGGGGCGGGGAAGAG
	OAS2p R	CCCAGAGCCAGGAAACTGAAAC
	ACTBp F	TTTCGCAAAAGGAGGGGAGAG
	ACTBp R	GCCGCTCGAGCCATAAAAG
Viral Genes (Species B)	7E1A F	TGATGAGTCACCTTCTCCTGA
	7E1A R	TGGCAGTTTCCGGGTACTAA
	14E1A F	CTCCTGAGATTCAAGCACCTG
	14E1A R	TTATTGTCTTGGCCGTTTCC
	7E1B F	ACCGTGCATATCGTTTCACA
	7E1B R	GAAAAGGCATCTGGTTCCAA
	14E1B F	ACCGTGCATATCGTTTCACA
	14E1B R	GAAAAGGCATCTGGTTCCAA
	7E2 DNA Pol F	CTTTTCTGGGGTGATGCAAT
	7E2 DNA Pol R	CTTTGGCAAGCAGCTAATCC
	14E2 DNA Pol F	GTGCGATTGGGTTTTTCTGT
	14E2 DNA Pol R	GTACTTGTCACCGCCCAACT
	7E3 F	ATTAAGTGCGGATGGGACTG
	7E3 R	CTGGGAGGCCATAGGTCATA
	14E3 F	GCTTCTTCAACCCGGATTTT
	14E3 R	CTTCCACCCAAGGGTTTTCT
	7E4 F	GTTATTTTGCCTCCCCCTTC
	7E4 R	GCAGCAGTTGGACTGTGAAA
	14E4 F	GTTATTTTGCCTCCCCCTTC
	14E4 R	ATGACCGTGATCCAGACTCC
	7Hexon F	CAAAGCCGTCACCTTACCAT
	7Hexon R	GTTCTGTCCCAGGTCGGTAA
	14Hexon F	CAGATGCTCGCCAACTACAA
	14Hexon R	AGCCATGTAACCCACAAAGC

Figure S17. List of qPCR primers used for gene expression and ChIP assays.
